# Creation and testing of the *Domiscore*—a tool to characterize the impact of housing on health and well-being

**DOI:** 10.1186/s12889-023-15451-y

**Published:** 2023-05-04

**Authors:** Aude Richard, Camille Bruat, Didier Febvrel, Fabien Squinazi, Jean Simos, Denis Zmirou-Navier, the members of the HCSP working group, Laurent Baillon, Valérie Bex, Pierre Deroubaix, Corinne Drougard, Pascale Estecahandy, Didier Febvrel, Nathalie Garrec, Ghislaine Goupil, Séverine Kirchner, Susanne Kulig, Laurent Madec, Nathalie Malou, Francelyne Marano, Laurent Martinon, Sophie Pamies, Jean Simos, Fabien Squinazi, Denis Zmirou-Navier

**Affiliations:** 1 Institute of Global Health, University of Geneva, Geneva, Switzerland; 2 HCSP (High Council of Public Health), Paris, France; 3 Université de Lorraine, Nancy, France

**Keywords:** Environmental health, Social Determinants of Health, Housing, Qualitative research, Interviews, Policy

## Abstract

**Background:**

Despite evidence of the major impact housing carries on health, many individuals still live in unhealthy dwellings. In France, the Domiscore has been proposed as a tool to assess the quality of dwellings with regard to their health impact, to allow for a better detection of unsafe housing and to improve dwellings. The aim of this paper is to present the method used to construct the Domiscore and test its relevance and usability.

**Methods:**

The Domiscore grid, inspired by the *Nutriscore,* consists of 46 variables—such as air quality, light or outdoor view. Each variable is scored on a four-point scale using in situ observation, mandatory diagnostics and open access data. The sum of each variable’s score results in an overall risk score for the dwelling. The Domiscore was tested in two phases.

During the first testing phase, 11 real estate professionals, health professionals and social workers used the Domiscore for on-site visits in different geographic areas of France. They then participated in a semi-structured qualitative interview.

The second phase consisted in a public consultation with diverse stakeholders such as public authorities, housing activists and social workers, using an online survey to collect their opinions on the Domiscore’s relevance, understandability and usability.

**Results:**

The Domiscore was tested on 28 homes. Variables completion rates were high irrespective of tester profile for all home visits (91%, SD = 4.7%). The mean time needed to fill in the grid was 1.5 h. The public consultation returned 151 responses. The *Domiscore* was deemed easy to understand, relevant, and rather easy to fill out. Most participants found the Domiscore useful for information gathering, awareness raising, detecting at-risk situations and agreed that it could contribute to enhance housing conditions. Its length was noted, although the inclusion of additional variables was also suggested.

**Conclusions:**

The results of this study suggest that the Domiscore is accessible to housing specialists and other professionals for the evaluation of a dwelling’s health impacts and the standardized detection of dangerous situations. The testing process allowed for improvements in the grid and training materials for future users.

**Supplementary Information:**

The online version contains supplementary material available at 10.1186/s12889-023-15451-y.

## Introduction

### Housing impacts on health

The impact of the living environment on health, well-being and productivity has been the subject of research and interventions for several decades [1]. Housing – defined as the dwelling itself and its close surrounding environment—carries diverse and complex effects on physical, mental and social health [2]. Additionally, the lifestyles of populations around the world has increasingly favored time spent indoors as populations migrated towards cities [3]: in some European countries and the United States, populations spend around 90% of their time inside buildings and transportation [4]. As a result, the public health community has grown more and more aware of the importance of housing as a health determinant, with interventions increasingly targeting urban planning [5]. Of note is also the additional effects of multiple exposures and a necessary global approach to risk reduction [6].

Housing is considered as a health determinant by the World Health Organization (WHO), as many factors in the living environment have been shown to influence health outcomes [5, 7, 8]. The following section summarizes key findings. Low indoor temperatures are usually the result of a combination of low outdoor temperatures and insufficiencies in the home’s envelope, insulation, or in heating; they can cause respiratory tract infections, worsen chronic lung diseases and increase the risk of cardiovascular adverse events [9]. Excess winter deaths due to cold housing have been estimated at 38 200 per year (12.8/100 000) in 11 selected European countries [6]. High indoor temperatures, on the other hand, usually result from high outdoor temperature, improper insulation and ventilation, as well as the absence of cooling systems, causing sleep disturbances, worsening of cardiovascular diseases, and adverse pregnancy outcomes, such as stillbirth or miscarriage. This health threat to the most vulnerable individuals will likely increase due to climate change in the future [7].

Numerous indoor air pollutants are found as gases or particles in the air and dust (volatile organic compounds and inorganic gases, particles, asbestos fibers, artificial mineral fibers, viruses, bacteria, molds, pet allergens, dust mites, etc.). Their effects on health span from discomfort (e.g. olfactory disturbance, eye irritation or drowsiness) to serious pathologies (e.g. respiratory allergies, asthma, domestic hypersensitivity pneumopathies, cancer) [10].

Mold is a very prevalent indoor air pollutant, estimated to occur in 10 to 15% of European homes [11] following an excess of humidity caused by poor ventilation, water damage, structural insufficiencies and cold surfaces (which cause condensation). Mold, while it can be invisible to the naked eye, can cause a wide array of illnesses, from rhinosinusitis, lung inflammation, sarcoidosis and toxic syndromes, to respiratory fungal infections, particularly in the immunocompromised [7, 12], and the development/exacerbations of asthma in children [13, 14].

Characteristics of the home’s close environment may also carry important impacts on health. Urban designs that discourage healthy eating and physical activity, such as lack of green spaces, footpaths and bicycle lanes, pollution, poor safety conditions, lack of access to healthy food, contribute to cardiovascular illnesses, such as obesity and diabetes, as well as poor mental and social health [7, 15]. Conversely, environments that encourage physical, social and cultural activities, and provide opportunities to purchase nutritious foods can have a positive impact on populations’ physical and mental health [7, 16–19].

Some populations (e.g. elderly, children, people with chronic diseases and socially disadvantaged) are more vulnerable to indoor environmental factors than others and therefore need special attention [20, 21]. Also, some of them tend to spend more time at home than the rest of the population, and thus are more exposed to health risks associated with poor housing conditions [22], in particular environmental noise and extreme high and low temperatures. Infants and children are also more susceptible to environmental toxicants, such as indoor lead, due to their developmental state [23]. Additionally, according to WHO [7], there is a 60% probability that a new home will be occupied at one point by a person with a functional disability; the issue of accessibility to housing is therefore essential.

### Housing in France

In France, despite considerable improvements over the last decades [24], the housing situation is still very difficult. According to the *Fondation Abbé Pierre*'s 2022 annual report on substandard housing, 2,819,000 people (around 4% of the population) were living in very poor housing conditions (no running water, shower, indoor toilets, kitchenette, or heating system, very deteriorated facade, or overcrowded household), with the Overseas Territories particularly affected by poor insulation and flooding [25].

Approximately 20% of French households complain of fairly frequent to frequent noise during the day, and this figure rises to 30% in collective housing. In large urban centers, complaints concern accessibility by car, street maintenance and air quality, especially in Paris, where neighborhood safety is also considered poor (5 times more often than in rural areas). In rural areas, complaints focus on the lack of public transportation (56% of households declare no access to public transportation or only to school transportation), and stores where to buy first necessity items (34%) [24, 26].

In order to broaden the definition of health considered in current French law and to better regulate the housing market with respect to the impacts of housing on occupant health, the French Ministry of Health began work in 2018 to update a foundational regulatory text that outlines minimum housing standards (for an overview of the French regulatory framework for safe housing, see Additional file 1: Appendix G in the supplementary materials). To support this work, the French High Council for Public Health (*Haut Conseil de la Santé Publique*, HCSP) 1 – a panel of independent experts—was asked to provide a tool that would integrate criteria to characterize a dwelling in terms of its positive or negative impacts on its occupants’ health and well-being.

### The Domiscore tool

The resulting tool called “Domiscore” consists of a multi-criteria grid which primarily aim at a global qualification of the habitat. It is to be implemented through the on-site completion of the grid by an evaluator, during a home visit and observation of the immediate environment, for a limited period of time, with the agreement and in the presence of the occupant.

The Domiscore is intended to be used by a wide range of people who, by virtue of their position, are entitled to visit homes. The profiles envisioned include municipal agents who evaluate potentially hazardous or unhealthy housing, social and medico-social workers, indoor environment advisors and all other professionals involved in housing (landlords, real estate agents, NGOs active on behalf of tenants, etc.). It is important to note that the tool was not built so as to be used by the occupant themselves, since it was felt that an external visit would allow for a better replicability within, and standardization between homes. The Domiscore was thus designed to be usable by a broad range of individuals with general knowledge of housing, without the need for specific technical skills or instruments of measure. An additional ask for the Domiscore is that it should take into account specific individual needs, the suitability of the home for vulnerable occupants (e.g. children, the elderly, disabled) or its potential for adaptation.

This article describes the process of the Domiscore’s creation and testing, on the one hand to assess how easily key housing characteristics could be converted into the Domiscore grid variables in a limited number of dwellings and, on the other hand, to investigate how housing specialists and stakeholders evaluated the usability and benefits of the proposed tool. Based on these results, the Domiscore was adapted.

## Methods

### The Domiscore tool, variable selection

The HCSP working group of public health experts selected variables for inclusion in the Domiscore through several iterations and meetings. Variables were selected based on risk and health promoting factors identified by the HCSP task force in a literature review described elsewhere [27]. The Domiscore questions were based on a review of the scientific literature (search of Scopus, PubMed and Google Scholar databases) and grey literature (inventory of agency and institute reports, such as World Health Organization (WHO) reference documents, books, thesis manuscripts, etc.), on the determinants of housing that can positively or negatively influence the health of occupants, without geographical or temporal restrictions. This literature review was complemented by interviews with French actors in housing, public health and social landlords. Emerging risks such as nanomaterials, electromagnetic waves, endocrine disruptors or connected objects and their impact on privacy were deemed outside the scope of the report and thus not considered.

**Fig. 1:**
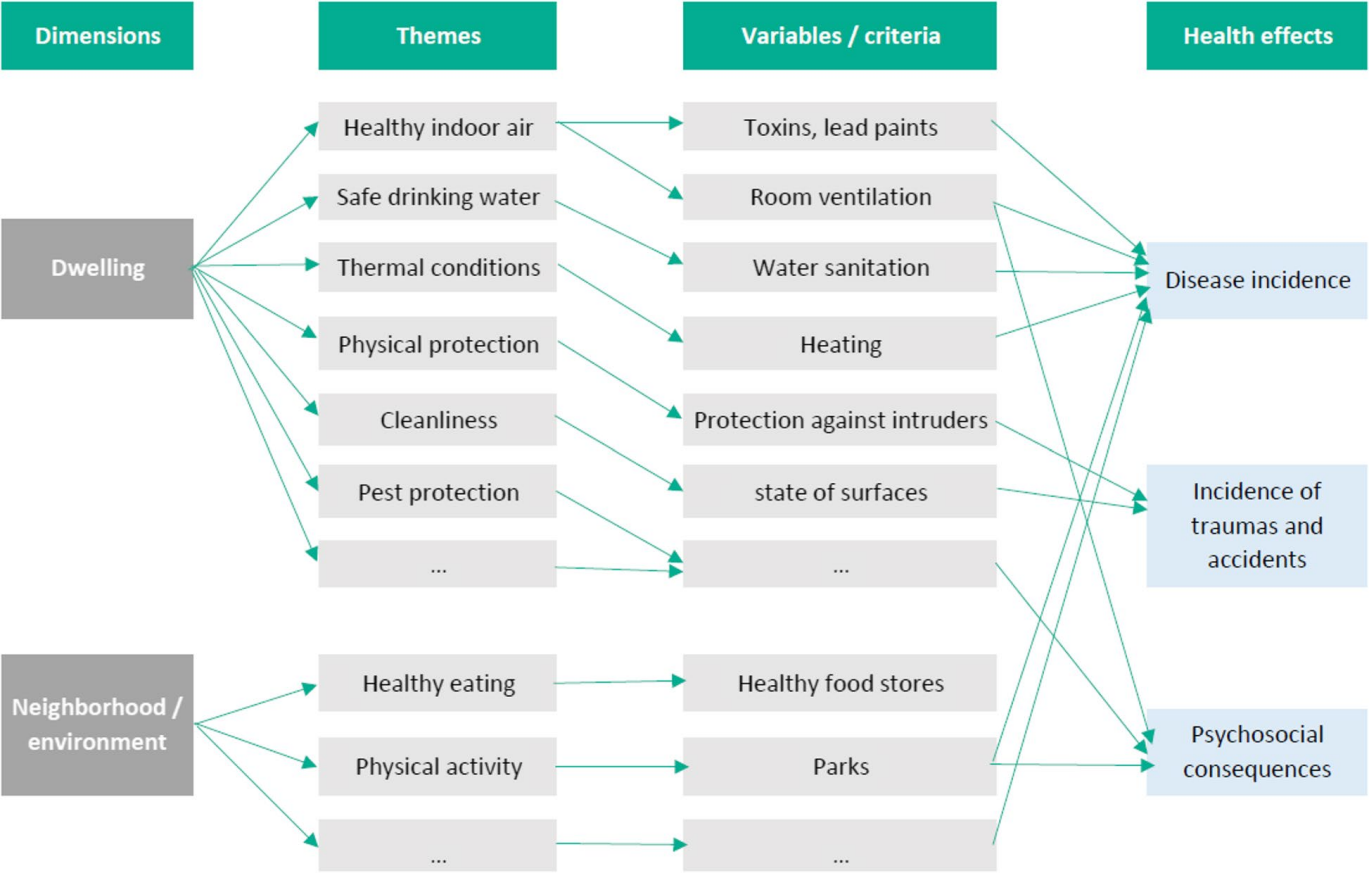
Representation of the rationale behind variable selection for the Domiscore (the themes and variables shown here are an illustration and not the final list)

In addition to their relevance as risk or health promoting factors covering the major themes identified as affecting occupant health, the criteria for selecting the variables were that they could be conveniently measured without the need for a measuring device, had little dependence on occupant behavior and assessor subjectivity, and were limited in number (see Fig. 1).

In practice, variables mentioned in the literature review report were successively discarded if they were:

- Too far from the objective of the Domiscore (education, family life, etc.),
- Too dependent on the occupants' behavior and/or not sufficiently dependent on the specific characteristics of the habitat (tobacco and cannabis consumption, presence of indoor plants, etc.)
- Too difficult to measure without specific devices and/or technical expertise,
- Likely to stigmatize the occupants or to be linked to the mode of occupation (overcrowding, clutter, animal crowding, etc.)

Initially, the grid consisted of a total of 47 variables clustered in 15 thematic categories, comprising 1 to 7 variables each. The thematic categories were the following: indoor air, exposure to environmental air and soil pollution, noise, lighting, pests, water, temperature conditions, indoor physical protection, waste, electricity, hygiene, accessibility, food supplies, outdoor view, and close environment conducive to exercise and socializing. Variables belonging to more than one theme were merged for the sake of conciseness. After testing, several variables were modified as explained in the discussion section of this article, and the grid now consists of 46 variables. The pre- and post-testing Domiscore grids are available in the Additional file 1: 1appendix.

### The Domiscore tool, scoring method

#### Information gathering

To evaluate a home using the Domiscore grid, several sources of information need to be mobilized, depending on the variable (see Additional file 1: appendix A: Domiscore grid January 31, 2020 approved version used for testing, Additional file 1: appendix F: Domiscore Grid with post-testing modifications, and Additional file 1: appendix B: Data sources to be used for filling out each variable):

- In situ observation of the habitat and its environment,
- Mandatory diagnostics (required by law),
- Open access data, notably from public platforms (georisques.gouv.fr, irsn.fr, etc.),
- The occupants’ reported experience and perceptions (for variables such as the discomfort caused by noise at different times of the day, evening or night).

Occupants were informed of the anonymous nature of the Domiscore and oral consent was obtained. No personal information on the occupant and no information allowing to identify a person or an address was recorded.

The scoring process is divided into two steps: (1) thematic scores, which characterize the habitat according to each of the categories considered in the score, and (2) an overall score, which characterizes the home in a summary form. The thematic scores allow inhabitants to quickly grasp the factors underlying the positive and negative health impact of their home, whereas a global aggregation of the sixteen thematic scores is necessary to make comparisons between homes or provide a summary view of a given territory.

#### Thematic score

Each variable is graded individually using a 4-level ordinal score going from 0 (most favorable) to 3 (most unfavorable), in order to find a balance between simplicity and accuracy of the assessment. The four grading levels are described with precision in the document, to ensure that the assessment is as reliable and standardized as possible. If information collection is made impossible by a lack of access to part of the home or the unavailability of mandatory diagnoses or open access data, the variable is not scored. In case of the unavailability of a mandatory diagnosis, this can be recorded in the document.

The thematic scores correspond to each of the highest (poorest) score obtained on at least one variable within each thematic category. This is based on multi-criteria decision-making approaches known as "aspiration levels" and allows for sectoral aggregation at the thematic level [29]. Thematic scores are ordinal variables that can be color-coded as follows: 0 = green, 1 = yellow, 2 = orange and 3 = red, on a similar model to that of the Nutriscore, which provides information about the nutritional quality of products in a simplified form [30]. The thematic assessment of a habitat, which characterizes its "profile," is therefore a series of colors tending more or less towards green or red, or white when not filled out, assembled in a table.

#### Global score

The global score is calculated as the arithmetic sum of the 15 thematic scores (16 in the latest version, see Additional file 1: appendix F). The choice was made not to weigh the different categories, because no strong scientific argument could be made to justify giving more importance to some variables than others. Thus, the global score varies from 0 (dwelling where all variables are judged as most favorable to health and well-being) to 45 (dwelling where all the themes present variables most unfavorable to health), 48 in the final version of the score.

In case one or more of the thematic categories was left blank, a penalty is applied to the global score, under the assumption that this absence of information is more likely to reflect a poor situation than the opposite. This penalty is calculated by dividing the sum of the scores of all completed thematic categories by the number of completed thematic categories. The calculated penalty is then added to the global score and the resulting sum is then rounded to the closest whole number.

Finally, the global score is classified into one of four equivalence grades that use the same quadri-chromatic scoring as the thematic categories. Knowing the bounds of the Domiscore, it is possible to qualify the intermediate scores by ranking them according to equivalence classes. This being a sorting problem (or β-problem or segmentation procedure, according to the theory of multicriteria decision-making) [31], a few standard profiles can be used to segment the range of possible scores according to the same 4 equivalence classes already used in the sectoral aggregation. If half plus one of all themes were scored as red (score = 3), it is reasonable to estimate that the sum of (7 + 1) × 3 = 24 should be placed in the range of values that will be part of the "red" equivalence class. We can therefore define the range of the red equivalence class as 24 to 45. Similarly, the sum of (7 + 1) × 2 = 16 must fall within the range of values that will be part of the equivalence class "orange" and so on, so the ranges of the Domiscore classes are as follows: 0–7 = green (dwelling favorable to the health and well-being of the occupants), 8–15 = yellow (dwelling with favorable factors for the health and well-being of the occupants), 16–23 = orange (dwelling with risk factors for the health of the occupants) and 24–45 (or more if penalties are applied) = red (dwelling that exposes occupants to a high health risk).

#### Accounting for occupant vulnerabilities

It is important for the Domiscore to acknowledge the interaction of housing variables with some individual vulnerabilities. Based on the scientific evidence regarding increased risks for vulnerable subgroups [8]—such as respiratory diseases among infants due to poor air quality, falls in elderly or disabled individuals due to physical housing characteristics -, a penalty was applied when some variables reached a certain score (1, 2 or 3) according to the vulnerability profile of the occupants, based on experts’ opinion. No provision was given to account for the severity of health effects.

**Fig. 2:**
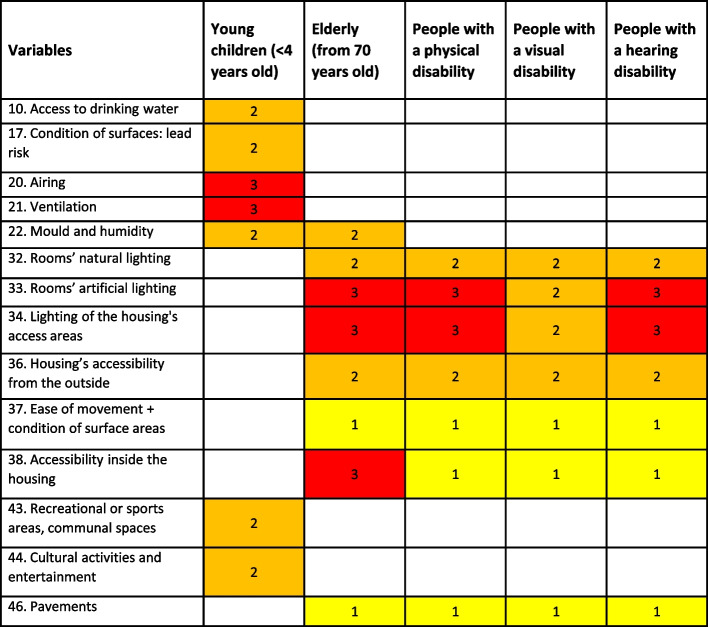
Penalties to be applied to variables according to the occupants’ vulnerabilities. A penalty point should be added to the variable from the rating in the table based on the vulnerability factors experienced by the occupants. Note: variable numbers correspond to the latest version of the Domiscore grid, after testing

The list of vulnerabilities includes: presence of young children (< 4 years old), presence of elderly people (over 70 years old), presence of people with a physical, visual or hearing disability, and involves 14 categories of housing characteristics (see Fig. 2). For example, variable "ventilation", where young children (< 4 years) are present, is downgraded by one point when it is given a rating of 3. Similarly, the same penalty is applied where persons with visual disability are present, if the variable “surface area” has a score of 1 or above.

#### Risk situations

The assessor must report any situation where the health and/or safety of the occupants may be endangered to the competent authorities (mayor's office, regional health agency or prefect) as soon as possible. The description of these variables includes a prompt to do so when rated 1, 2 or 3 (depending on the variable) (see appendices A and F).

### Testing

The objectives of the testing were multiple: 1. to collect specialist opinions on the Domiscore and its potential uses; 2. to ensure that the tool can be understood and used by a wide range of professionals and that the variables and score scales are relevant; 3. to identify any difficulties encountered when filling in the grid.

This testing was conducted in two phases. A first phase assessed the ease to inform the Domiscore grid variables and the scoring method by a variety of professionals in a real-life situation in September 2019. Then the HCSP launched an online public consultation in the first half of 2020, with the goal to gather laypeople and specialists’ opinions on the grid’s comprehension, potential for implementation and the relevance of the chosen variables.

### Test of the scoring method

#### Participant recruitment

Under the supervision of the *Espacité* and *Planète Publique* consultancies [32], the test phase, which was conducted from September 3^rd^ to 23^rd^, 2019, mobilized 11 individuals from different regions of France and professional backgrounds, specialized or not in the housing field. These evaluators were identified by members of the expert panel. Table 1 shows the profiles of the participants and a breakdown of the most prevalent profession, housing technician.

**Table 1:** Profile of the users involved in the test phase

**Region**	**Number of participants**
Auvergne-Rhône-Alpes	5
Île-de-France	1
La Réunion	1
Martinique	2
Nouvelle-Aquitaine	1
Provence-Alpes-Côte d’Azur	1
**Total**	11
**Profession**	**Number of participants**
Housing technician	6
Real estate / lease management agent	2
Social worker	1
Childcare assistant	1
Indoor environment consultant	1
**Total**	**11**
**Detail of housing technicians**	**Number of participants**
Indecent housing operations manager	2
Municipal employee, housing services	2
Technician specialized in energy efficiency and indecent housing monitoring	1
Lawyer certified in housing decency reports	1
**Total**	**6**

#### Participant training

From September 3 to 5, 2019, all evaluators followed a two-hour live training session by videoconference or telephone, supplemented with a document functioning as a guide to filling in the Domiscore grid. The aim of the training was to present the Domiscore grid, the test procedure and the data gathering method, specifying the sources of information to be used for each variable. The training materials are available online [28].

#### Data collection

After the training and until September 23, 2019, the professionals put the Domiscore grid in application in homes to which they had access in the context of their work. Each tester had been given an objective of visiting three homes. The evaluators filled out 28 Domiscore grids (2 to 3 grids by participant). Two of the grids were filled out in the same home by different testers at different times, as a preliminary step to assess reproducibility. The grids were filled out without reference to the location’s address or the occupants’ identity. Data recorded on the dwelling were the following: collective / individual housing, urban / rural area, overseas, social housing, owner-occupant / tenant and region. The aim of this test was not to be representative of the diversity of French homes; rather the evaluators were asked to look for homes of different profiles to test the Domiscore grid in different housing settings.

From September 23 to October 1^st^, 2019, individual semi-directive interviews were conducted by telephone with each tester, to record the following data: time needed to complete the Domiscore grid, including preparation before the visit, time spent on site, and after the visit; any difficulties encountered with one or more variables, such as in understanding the variable, scoring, etc.; any unavailable information or inability to observe certain variables due to housing specifics; opinions on the relevance of the scoring variables, redundancies, lacking dimensions; the use, if any, of specific professional skills related to the evaluator's profession (which would diminish the universality of scoring); effectiveness of the provided training and any difficulties in the process in relation to the occupant.

#### Data analysis

Based on the collected data, the *Espacité* consultancy group was able to provide HCSP with a detailed report on the results of the on-site evaluation of the Domiscore grid in real housing. This report focused specifically on (i) the added value, relevance, and usefulness of the tool in the pre-identification and identification of problematic dwellings; (ii) the potential types of dwellings and/or family situations where the tool could be most useful; (iii) the receptivity of the actors, and possible target professionals; (iv) blocking points and limitations; and (v) changes to be made to the tool.

### Public consultation

The public consultation was first envisioned as a two-step project, with the first step being the gathering through an online questionnaire of laypeople and specialists’ opinions of the ease of comprehension of the Domiscore, and the second step being a real-life test with professionals using the Domiscore during actual homes visits within their regular work activity.

However, due to the epidemic of Covid-19 and a national lockdown that started on March 16th, 2020, in France [11], some aspects of this plan had to be changed. The site visits were dropped and the consultation was refocused on acquiring expert opinions on the Domiscore. A first sample of about thirty people were consulted from April 9 to April 14 about whether or not the consultation should be launched at a broader scale given the epidemic context, with on one side the ethical implications of launching it while many professionals were struggling in the handling of the epidemic, and on the other side the negative impact of poor housing conditions being worsened by the lockdown [12]. Following positive feedback, the public consultation was launched on April 15, and remained open until June 30, 2020.

#### Participant recruitment

Participants were recruited through emails sent on the 10th of April for the small-scale consultation and 14th of April for the larger-scale consultation. A list of email addresses of a nationwide set of organizations and individuals who carried out roles related to housing and/or public health was built, using internet searches and a mailing list of a previous consultation [33]. The mailing list in its final version included 1 266 contacts and 35 email addresses used for the test period were extracted from this mailing list. The distribution of professionals in both mailing lists is presented in Additional file 1: Appendix C. In addition to the emails, announcements of the consultation were published on different health networks’ websites, newsletters or LinkedIn accounts.

#### Participant profiles

A total of 152 responses to the anonymous online questionnaire were recorded. One observation was deleted due to missing data in professional title, resulting in a total of 151 participants. Two observations had missing data in one of the multiple-choice items (question 5 and 6, respectively), which was replaced with the least favorable response choice (not relevant and difficult, respectively).

The distribution of participant occupations is presented in Table 2. Of the 151 participants, a majority (52%) were civil servants from state services (e.g., Regional Health Agencies), as well as from local communities. Associations working to combat substandard housing, for the rights of families or for health accounted for 15% of participants, while social or medico-social workers accounted for 9% and 7% were indoor environment advising consultants. The other respondents were real estate professionals, private citizens, academics, elected officials, social landlords and other individuals. None of the invited private landlords and governmental health agency workers responded to the survey.

**Table 2:** Professional profiles of the consultation participants

Professional category	N(%)
State service	39(26%)
Housing professional for local community	39(26%)
Association for decent housing, family rights or health	22(15%)
Social or medico-social worker	14(9%)
Indoor environment advising consultant	11(7%)
Real estate professional (real estate agent, architect …)	8(5%)
Citizen with no specific professional profile	5(3%)
Academic research	4(3%)
Other	4(3%)
Elected official	3(2%)
Social landlord	2(1%)
Private landlord	0(0%)
Health agency (Anses^a^, Santé Publique France)	0(0%)
**TOTAL**	**151**

^a^Anses: French Agency for Food, Environmental and Occupational Health & Safety, Santé Publique France: French Public Health authority

A few participants wished to take part in the survey as private citizens, others did not fit into any of the proposed professional categories and were grouped under the category "Other"; these were an agent of the ASN (*Agence de Sûreté Nucléai*re), a health/housing engineer, a health consultant and an agent of the WHO.

#### Data collection

The HCSP’s website’s home page presented the public consultation and the different steps to participate. Consultation materials were available online including the Domiscore grid (see Additional file 1: appendix A), executive summaries of its preliminary report in French and English [34], guidance materials, a video tutorial, as well as the consultation questionnaire (see Additional file 1: Appendix D).

Data gathering included both a quantitative and a qualitative side. Quantitative data was gathered using 7 multiple-choice questions with between 5 and 8 answer choices (one of them being “other” and allowing free-text entry) or 4-point Likert-type scales to measure features such as ease of understanding (Easy, Rather easy, Rather difficult, Difficult), relevance and ease of filling-out. Comment boxes were provided for each question, as well as at the end of the questionnaire, to record qualitative data. The body of each question is provided in the results Sect. "Responses to multiple-choice questions" of this article.

#### Analyses

The quantitative analysis had two main objectives: 1. To describe the characteristics of the individuals who chose to participate; 2. To gather participant responses to several questions regarding the potential applications of the Domiscore, its ease of understanding, as well as the relevance of chosen variables. Data was analyzed using the RStudio software. Missing data was handled according to the following procedure: any observation with missing data in the professional title was deleted. Missing data in the open response fields or email address were kept as they were. Missing data in the multiple-choice answers were replaced with the least favorable response in terms of the Domiscore’s evaluation.

The qualitative analysis had the following objectives: 1. To gather participant opinions on the practical implementation of the Domiscore grid; 2. To find out about potential difficulties in filling out the grid. 3. To gain feedback from professionals who may be involved in the implementation of the Domiscore in the field of housing, in order to further adapt and improve the tool and its applicability in a real-life context. The comments received through email during the period of the consultation were included into the analysis. Comments were coded and summarized according to themes using Microsoft Excel (Microsoft Corporation).

## Results

### Scoring method and grading test

#### Profiles of the evaluated homes

Evaluators conducted 28 visits of 27 homes (one home was visited twice). Most of the visits (71%, 20/28) took place in urban environments and in the French metropolitan area (68%, 19/28), as opposed to overseas (Martinique and Réunion islands). Most (68%, 19/28) of the evaluations were done in collective housing, rather than individual houses. Privately rented homes were the most frequent (46%, 13/28), followed by social housing (32%, 9/28) and homes lived in by their owner (22%, 6/28).

#### Evaluator feedback

The semi-directive interviews conducted with the evaluators showed a mean completion time of the Domiscore survey of one and a half hour (91 min), with a mean time of 29 min spent before the visit, 48 min during the visit and 17 min after the visit, to complete the variables.

The mean completion rate of the grid was 91% (SD = 4.7%). The reasons invoked by the participants for not filling out the remaining 9% variables were first of all the unavailability of the data (5%), as in the case of mandatory diagnoses or online sources. 2% was due to the complexity of the on-site evaluation, 1% to non-explicit descriptions of the variable grading process and the remaining 1% to other nondescript reasons.

There was no apparent link between occupation type and variable completion, which suggests that the Domiscore is suitable for use by a wide range of professionals. The scoring test also showed no difference in time to completion and inhabitant acceptance according to tester occupation.

Additionally, the analysis of the completed grids made it possible to verify the absence of any variable likely to systematically degrade the corresponding thematic score.

The preliminary training (1h30 on average) was judged globally sufficient by the participants. However, the following difficulties were pointed out by several testers: the difficulty to consult online data on soil and air quality (several sites, varying according to region); the unavailability of some diagnoses (asbestos for example); several testers stated that they found it difficult to score variables that required their own assessment supplemented by exchanges with the occupant, for example, the perception of indoor/outdoor noise.

#### Amendments made to the grid

Several amendments were made to the grid and the training materials based on feedback given by the evaluators during the interviews. These included better wording for the descriptions of variable scoring. Two participants also suggested the addition of an "access to healthcare" variable, which was included as variable 39 "access to basic services". Beyond these needs for precision and clarification, no gaps in the grid or unnecessary variables were identified by the evaluators.

Variables relying simultaneously on occupant feedback and assessor direct observation, such as in the case of perceived nuisances in the home, testified to the difficulty of making a balance between multiple sources of information. The training material was amended to clarify the evaluator's role in the scoring of variables for which the discomfort felt by the occupant may take precedence over the evaluator's assessment, which is necessarily limited at the time of the visit.

Most of the non-filled-out variables resulted from the unavailability of mandatory diagnostics or difficulties consulting public online data platforms. On this last point, it was decided to add more explanations to the training materials to facilitate the use of these platforms. For the variable *radon*, the possibility to perform measurements on site instead of using the public database was included to respond to this problem.

### Public consultation

#### Participants

A total of 151 participants answered the survey, with a participation rate of 12%. (151/1266). 75% of the participants who started to fill in the questionnaire went all the way to the end of the process by completing the survey.

Participation rates were also uneven across professional backgrounds. Two thirds of participants are professionals in housing and health from local authorities or state services and associations fighting against substandard housing. These professionals, for the most part, have technical knowledge of housing and experience of substandard housing for the most part.

We found it interesting to compare participation rates in different professional categories with recruitment efforts (detailed in Additional file 1: appendix C). The majority (52%) of participants were state service or housing professionals working at the local level, which is mirrored in the communication effort where these categories were also the most represented, with 629 out of 1266 e-mail addresses (around 50% of the mailing list, see Additional file 1: Appendix C), and the most likely to be reached through publications within health networks and their newsletters. On the other hand, private landlords were only targeted through 2% of the mailing list (27 e-mails sent), which may be one reason for their non-participation.

However, there are also discrepancies between communication efforts and response rates. For example, social landlords, who represented 17% of the mailing list (209 e-mails), only account for 1% of the final sample. Elected officials, despite being pretty well represented in the mailing list with 208 emails (16%) addressed mainly to individuals but also targeting various of their professional networks, only accounted for 3% of the final sample. Conversely, associations fighting indecent housing, as well as social or medico-social workers were targeted by 1% (*N* = 10) and 3% (*N* = 34) of sent emails, respectively, and had relatively high participation rates of 15% and 9%, respectively.

#### Responses to multiple-choice questions

**Fig. 3:**
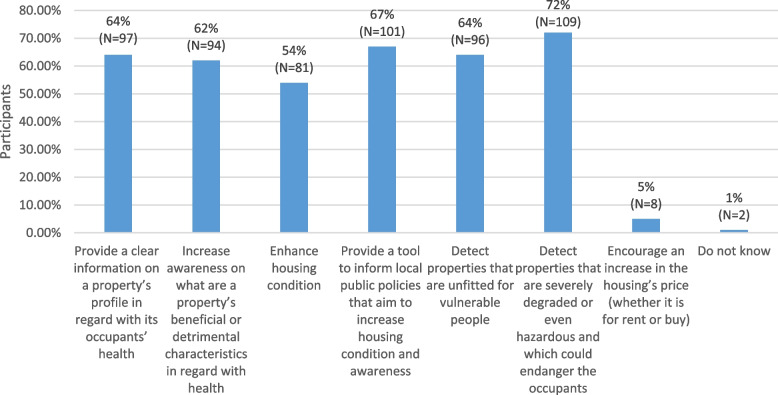
Response rates to the question “According to you, what advantage(s) and disadvantage(s) does a tool that characterizes a housing in regard with the occupants’ health represent? (Whether it is the Domiscore or another tool you may know)”

**Fig. 4:**
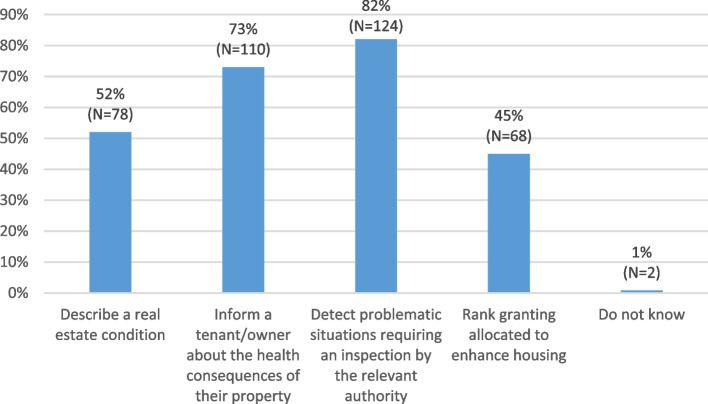
Response rates to the question “According to you, what application(s) of the Domiscore grid could be conducted by housing or city planning professionals?”

**Fig. 5:**
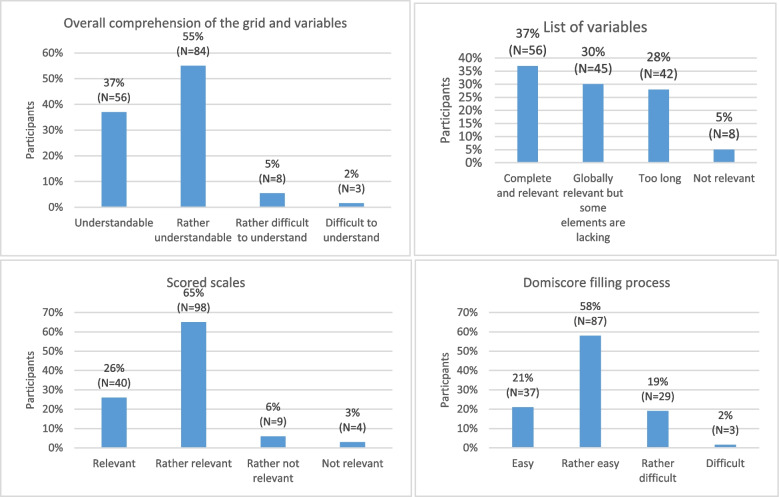
Response rates to the questions 3 to 6, respectively "How would you rate the structure and the different variables of the Domiscore grid?", "How would you rate the variables selected within the Domiscore grid as factors influencing health?", “How would you rate the scaled score assigned to each variable of the Domiscore grid?” and "How would you rate the Domiscore grid’s filling process?"

Figures 3, 4 and 5, shows the responses to the multiple-choice questions included in the consultation. A summary of participant responses to all closed and open-ended questions can be found in Additional file 1: Appendix E.

Overall, the structure, variables and scores of the grid were judged rather understandable and relevant and the filling out process rather easy. However, around one fourth of respondents found the list of variables too long. Most participants had a very positive view of the Domiscore, since they answered yes to the questions of whether it could help provide information on properties in regard to occupant health, raise awareness to the health effects of housing, inform public policies, as well as detect risk situations and ultimately potentially enhance housing conditions. Very few participants thought it could participate in raising rent or sale prices, which is encouraging for the implementation of the tool.

In terms of the applications of the Domiscore by housing or city planning professionals, most participants thought they could use the tool to describe the condition of a property park, signal problematic homes to the authorities, as well as inform tenants/owners on the health impact of their home.

#### Responses to open-ended questions

The most frequent comments in response to each question are summarized in Additional file 1: Appendix E. Participants underlined the potential of the Domiscore for awareness and health promotion. Respondents proposed the following uses: to make a tenant or owner aware of the work needed to improve the impact of their home on health, to support a request for renovation work, to evaluate the effects of a renovation or rehousing on health, to help design housing that better considers the notion of health in all its dimensions, as well as for occupants to use the Domiscore grid to identify the health impact of their housing and to help them detect problematic situations.

Generally speaking, the grid was perceived as too long. In particular, the prior consultation of public online data and diagnoses, as well as the in-situ observation of the neighborhood were perceived as time-consuming, although useful. Concerning the regulatory diagnoses, several participants specified that some documents were often missing, confirming the experience of the grading testers. The complexity of certain terms was pointed out and the introductory section "housing characteristics" was deemed not clear and detailed enough.

Several people stressed the need to better highlight potential imminent physical risks, clarify the procedure and add more variables to report to authorities in case of a score of 2 or 3. It was also mentioned that the scoring should not be interrupted even in case of alert to the authorities.

Some participants mentioned that the tool favored dwellings located in urban areas and penalized dwellings located in the countryside, citing the "public transportation" and “bicycle path” variables as examples where a rural dwelling might not have those, despite being low risk for health.

Many participants, especially State services and housing workers at the local level, pointed to the inclusion of the residents' assessment, for example, for noise and light pollution, temperature and the proliferation of pests, as subjective and prone to biases. Others, in particular social workers, welcomed the consideration of the inhabitants' opinions.

Finally, some participants expressed concern about the impact of the Domiscore on the occupants of the homes being assessed, in particular the anxiety that such a list of risk factors could provoke and confusion about the score received, as occupants could interpret their dwelling as unhealthy when the issue merely dealt with elements of comfort.

#### Amendments to the grid

Based on the many participant comments, the working group felt it was appropriate to amend the grid in several ways. It was agreed that the most relevant modifications of content and score scales suggested by participants should be considered, but that the Domiscore grid should not be made any more complex or detailed than it already was. In fact, many participants thought the tool was too technical to be filled by workers outside of the housing field.

To respond to this, the scoring scales were simplified, clarified and made more objective. For example, in the case of heating and cooling, the nature of the "natural" cooling and heating systems were specified (thermal insulation, window protection, window shutters, possibility of night ventilation, Nordic well, air-ground heat exchanger, etc.). Regarding drinking water, the reference to the "quality" of drinking water was removed since evaluators would in most cases not have the tools to assess this on site. Finally, the notion of flow rate was also removed because the variable overlaps too many criteria: access to drinking water and flow rate.

Clarifications regarded several variables, of which a few examples are given here. In the variable mold, a more precise description of the surface was included. In the variable air pollution, changes consisted in the addition of a more precise air quality scale and an indication that the air quality index should be considered over time (one year), since there can be variations and the effects of chronic exposure are what is of interest here. In the variable room size, a more complete description of the room dimensions was provided. For the sake of clarity, the question of pesticides was separated from outdoor air pollution, as an additional variable. Finally, the possibility to capture housing typology was added, such as studio, one bedroom, etc., as well as occupancy patterns other than owner-occupied and rental, and private vs social housing.

Since another frequent remark was that the tool was too long, the variables “ease of cleaning” and “adequate kitchen space” were removed. The first one because it was deemed non-essential and difficult to evaluate, the second because it is evaluated through other variables (electricity, rooms sizes, access to drinking water, access to hot water) and because the furnishing of a kitchen is inherent to the owner or the tenant and not to the dwelling itself.

Several participants having called for a broadening of the notion of imminent risk requiring reporting to the authorities, this requirement was extended to nine variables and the threshold grade for reporting was lowered on several occasions. Also, seven thematic categories were moved to the beginning of the grid, so that the most urgent threats to health are immediately identifiable.

It was also pointed out that the evaluators were invited to stop filling in the Domiscore grid if reporting to the authorities was necessary. However, this potentially removes these dwellings from a housing stock evaluation conducted in a given territory, thus biasing the evaluation by overvaluing the stock. Therefore, it was decided to invite evaluators to continue filling out the grid even when risk situations are identified.

In an effort to make the tool more adaptable to a rural context, the “public transportation” variable was changed to "access to basic services” (Physician, drug store, school, post office), as many villages do not have the capacity for public transport and this variable was intended to capture access to basic services, no matter the means of transportation. The notion of safe roads was added to the “bicycle path” variable, so that country roads without bike lanes (as many roads in the countryside), but are safe to bike, could be scored accordingly.

The results displayed as a score of risk, where 0 is the lowest risk and 3 is the highest risk, seems somewhat counter-intuitive, though correct in this instance. To clarify this, the notion of 0 being the best grade and 3 being the worst was added at the top of each thematic category section. The latest version of the Domiscore, dated November 2020, is included in Additional file 1: Appendix A and freely accessible on a dedicated webpage [28].

## Discussion

Overall test results show that the score can be applied by a wide variety of professionals, within a reasonable time. The structure, variables and scores of the grid were rather understandable and relevant and the filling out process rather easy, although relative difficulty in obtaining mandatory diagnoses and publicly available data, constitutes an argument in favor of using the grid at targeted times like renting, selling, etc., when similar diagnostics are required.

Most participants thought the tool could have multiple uses (provide information on properties regarding occupant health, raise awareness, inform public policies, and detect risk situations and ultimately enhance housing conditions), and very few thought it could participate in raising rent or sale prices, which is encouraging for the implementation of the tool. In terms of the applications of the Domiscore, most participants thought housing or city planning professionals could use it to assess a property park, signal problematic homes to the authorities, and inform tenants/owners about the health impact of their home.

Also, different professional categories showed different interpretation of the impact of assessor subjectivity and the reliance of certain variables on resident perception. State services and housing professionals at the local level tended to be concerned about a lack of objectivity of the scores, while social workers were more likely to advocate for an even greater integration of the residents’ opinions. Public service professionals are used to working with tools to evaluate buildings likely to be declared insalubrious, which involve very objective criteria and are not used in a health promotion approach. This may explain their often-unfavorable reaction to the inclusion comparatively more subjective elements in the Domiscore, such as scoring the of the close environment and the views of occupants. Additionally, since the tool was created to raise awareness about housing elements that might impact not only health but also well-being, it is inevitable that the score be somewhat subjective. Therefore, one should keep in mind that the score attributed to one dwelling might differ slightly from one resident to another.

Finally, the Domiscore should be universally applicable, i.e., to all types of housing regardless of occupants’ characteristics. Since it is intended to qualify habitats which occupation evolves over time, it does not account for occupant behavior. This in no way implies that occupant behavior is considered to play a negligible role in the appearance or aggravation of health risk factors. Occupant behavior is accounted for in other initiatives, for example in the work of interior environment advisors (*Conseillers médicaux en environnement intérieur*—CMEI, [35]).

### Limitations of the testing phases

This article describes the creation and testing of the Domiscore tool. As such, the testing did not implicate a large sample of participants, which is of course a disadvantage in terms of statistics. However, the overall results are encouraging in opening the way to further testing in real-life situations.

#### Grading test

Diverse professionals put the Domiscore grid in application on their own, in homes to which they have access in the context of their work. This means that the environments in which the tool was used and the testing process was neither randomized, nor standardized. On the other hand, the process used in the scoring test is more realistic, in that visits will most likely be conducted by professionals used to visiting certain neighborhoods and buildings and not being accompanied in the Domiscore implementation.

Another limitation of the grading test is the lack of inter-operator reliability testing. This would have necessitated the comparison of the score given to each variable of Domiscore grids filled out by different observers on the same dwelling. At the time this did not seem feasible since participants were located in different regions of France and overseas. Indeed, the scope of this study was to assess the feasibility of the Domiscore tool on the ground and whether it could be used by professionals from diverse backgrounds, as well as to gather improvement suggestions. However, this is an important and necessary criterion for implementation and will necessitate further testing.

#### Public consultation

The first limitation of the public consultation is that the global participation rate was low and uneven across professional backgrounds, a selection bias in favor of professionals in housing and health from local authorities or state services and associations fighting against substandard housing, with low participation rates and thus underrepresented opinions from elected officials, social workers, landlords and researchers. Also, the uneven distribution of professional categories led to a limitation in the ability to detect possible differences in responses due to low statistical power. It would be useful to ascertain the reasons behind the low participation of certain professional groups, since this could be an indicator of their future participation in the Domiscore implementation.

We formulate several hypotheses regarding this uneven participation: First, the recruitment process is likely to have played a role in participant distribution, as shown in the results part of this manuscript. However, some discrepancies between email targeting and participation suggest that intrinsic motivating factors might be at play, such as scope of understanding and interest in the subject, as well as potential for benefitting from or feeling constrained in their work by an eventual implementation of the Domiscore. Finally, Covid-19 meant that some professionals, like elected officials, were devoting most of their time to the management of the epidemic. As another limitation, it is worth mentioning that the multiple-choice items used in the quantitative part of the questionnaire is by design likely to have oriented participant answers to some extent.

### Limitations of the Domiscore

#### Synthetic qualification of a habitat

The Domiscore aims to give an appreciation of a multiplicity of factors and complex problems through a classification simple enough for a wide range of non-specialists to understand and use. To this aim, a balance was sought between exhaustiveness on the one hand and clarity and conciseness on the other hand. The grid therefore uses a summary of all factors related to housing and health. The choice of variables largely independent of the occupants also makes the tool easier to use and deploy, and avoids the use of sensitive identifying information.

#### Incomplete standardization of publicly available data on different territories

Several variables are scored according to indices available online, such as: protection against natural and technological hazards, radon, air pollution, outdoor soils (variables 4, 5, 23, 26, 28). For natural and technological hazards, the availability of data is dependent on the entry into force of the corresponding hazard prevention plans, which, in turn, partly depends on local decisions. Although these sources will tend to become uniform in the long run, the varying degree of progress of these risk prevention plans may lead to differences in scores depending on the territory, which can bias comparisons at a regional or national level. On the other hand, it does not lead to differences in rating within the same territory.

#### Impact of missing information on scoring

For variables requiring the consultation of a compulsory diagnosis, when it is unavailable, a box can be checked to indicate the impossibility to score the corresponding variable. The categorical scores are then calculated based on the variables that could be filled in, disregarding the ones that could not. It is therefore possible for some habitats to have a less degraded score because of the absence of a diagnosis than if a very poor diagnosis had been provided. The availability of mandatory diagnostics is therefore an important factor in the robustness of the Domiscore.

#### Consideration of the occupant's opinion

Some variables cannot be exclusively assessed by the evaluator and therefore require asking the occupant about perceptions, such as perceived discomfort relating to noise and visual nuisance, for example. This however, means that factors like personal values, preferences and cultural representations can influence the characterization of the habitat. Detailed descriptions of the variables with examples, as well as evaluator training and guide documents, make it possible to limit but not totally eliminate the bias introduced by questioning the occupants. Still in the case of nuisances, it is also possible to compare the level of discomfort declared by different occupants of a same building or neighborhood, keeping in mind that it is a subjective matter that can be experienced differently between individuals.

### Future perspectives

The Domiscore can serve as a guide to describe the state of housing on the scale of a territory of any dimension (district, metropolitan area, city block, etc.) and to inform housing improvement policymaking. Communities (mayor offices, regional health agencies, etc.) have agents regularly inspecting homes in order to detect insalubrity; they could have these systematically use the Domiscore on a representative sample of their dwellings. Aggregating the global scores of the investigated homes will give a picture of the quality of housing at the territory level. By systematizing this approach throughout their jurisdiction, these communities will gain knowledge of the spatial and social distribution of housing quality. Thus, beyond inventory, the Domiscore is aimed at promoting continuous improvement of housing from a public health point of view. It can also trigger reports and specific technical diagnoses when the rating of certain variables raises concern, in particular when the housing presents a risk of insalubrity, endangerment, or indicates social, sanitary or medical situations that require specific attention from the authorities. In addition, the Domiscore is a health promotion tool, empowering inhabitants by giving them knowledge about factors in their home that can negatively and positively impact their own health and wellbeing.

The HCSP proposed that an evaluation of the tool be scheduled three years after implementation on the basis of which changes could be recommended. This evaluation could be based on data provided by regional health agencies, cities or municipalities, which could submit an annual report on the visits carried out in their territory to the Ministry of health. The methods of implementation of this evaluation are still under discussion.

## Conclusion

With the Domiscore, the High Council for Public Health aims to initiate a global approach to the factors contributing to a healthy home, in a health promotion perspective. As further evidenced by the Covid-19 epidemic, good indoor conditions such as IAQ, are crucial not only to limit the transmission of the virus, but also for their general impact on human health and wellbeing [36].

The Domiscore tool was developed using a scientific review of the evidence, and testing suggests that it is accessible to a range of different professional groups, to be used for the standardized identification of at-risk situations and for health promotion, taking in consideration factors that have a positive impact on the health of inhabitants.

Further testing of the Domiscore in real-life situations and user feedback will be precious in the process of implementation, which should be iterative, as experience gained in the field continuously informs improvements and updates. Also, since the Domiscore aims to mobilize scientific evidence into policy, it is intended to change and evolve as knowledge about the link between housing, health and wellbeing grows in the future.

## Supplementary Information

**Additional file 1.**

## Data Availability

The datasets used and/or analysed during the current study are available from the corresponding author on reasonable request.

## Footnotes

1 The High Council for Public Health (HCSP) in France is an independent public body that advises ministers and parliamentary committees on public health policies. It is made up of nearly 80 independent experts, including medical specialists, college teachers, university hospital physicians, and researchers in public laboratories.
