# Supplementary material for: Creation and testing of the Domiscore—a tool to characterize the impact of housing on health and well-being
Source: BMC Public Health. 2023 May 4;23:815. doi: 10.1186/s12889-023-15451-y (PMC10157125; doi:10.1186/s12889-023-15451-y)
Supplement: Supplementary file 1 — Additional file 1. [file 12889_2023_15451_MOESM1_ESM.docx]

**Supplementary materials**

1. **Domiscore grid, January 31^,^ 2020 approved version used for testing**

**Domiscore grid**


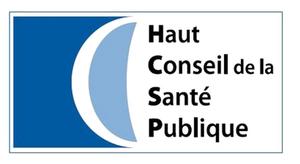


**French High Council for Public Health**

(provisional form; amendments are expected fall 2020)

Any reproduction and use of the Domiscore grid should mention its origin: *French High Council for Public Health*. Its text may be amended by the HCSP only.

***Housing’s characteristics***

*Collective housing* ☐ *Individual housing* ☐

*Urban area* ☐ *Rural area* ☐

*Overseas* ☐

*Social housing* ☐

*Owner-occupier* ☐ *Tenant* ☐

*Region: .....................................................................................*

| **Indoor air** | | | | |
| --- | --- | --- | --- | --- |
|  | **0**  (best-case) | **1** | **2** | **3**  (worst-case) |
| 1. Airing | ☐ | ☐ | ☐ | ☐ |
|  | *There are easy-to-open windows in all living areas* | *There are windows but they cannot be opened properly or a slight obstruction limits the extent to which they can be opened in at least one of the living areas* | *There are windows but they cannot be opened properly or a major obstruction makes them very difficult to open in at least one of the living areas* | *No windows in any or some of the living areas* |
| 2. Ventilation | ☐ | ☐ | ☐ | ☐ |
|  | *There is functional continuous mechanical or natural ventilation (operationally controlled mechanical ventilation system, securely placed base-level and above-level air inlets) in all rooms* | *Absence of ventilation in one of the washing areas (bathroom, WC, kitchen)* | *Absence of ventilation in at least two washing areas* | *Absence of any ventilation* |
| 3. Mould and humidity | ☐ | ☐ | ☐ | ☐ |
|  | *No humidity (excluding window or door seals)* | *No humidity but traces of mould (excluding window or door seals)* | *Presence of mould in washing areas only, or across less than 3 m² for all rooms in the housing quarters (excluding window or door seals)* | *Presence of mould across more than 3 m² for all living areas in the housing quarters (excluding window or door seals)* |
| 4. Radon  [See the map here](https://www.irsn.fr/FR/connaissances/Environnement/expertises-radioactivite-naturelle/radon/Pages/5-cartographie-potentiel-radon-commune.aspx) | ☐ | ☐ | ☐ | ☐ |
|  | *Municipality referenced 1 by the IRSN* (low risk)* | *Municipality rated 2 by the IRSN (potential risk)* | *Municipality rated 3 by the IRSN (high risk) but housing located on an upper floor or system enabling evacuation of radon in the sub-soil* | *Municipality rated 3 by the IRSN (high risk) with no known evacuation system + housing that is underground or semi-buried, on the GF or 1st floor$* |

| 5. Asbestos  If there is no technical diagnosis or if unknown, do not complete but  tick here ☐ | ☐ | ☐ | ☐ | ☐ |
| --- | --- | --- | --- | --- |
|  | *No asbestos in the housing or communal areas* | *No asbestos in the housing quarters, but presence in the communal areas with periodic assessment required* | *Presence of asbestos in the housing quarters and periodic assessment required* | *Presence of asbestos in the housing quarters and removal recommended or required* |
| 6. Carbon monoxide | ☐ | ☐ | ☐ | ☐ |
|  | *No system using a carbon-based fuel (wood, pellets, coal, gas, oil, etc.)* | *There is a gas cooker or*  *there is another carbon-based fuel-fired appliance with good ventilation*  *+ contract bearing the appliance's maintenance and/or the flue sweeping frequency* | *There is an appliance (other than a gas cooker) using carbon-based fuel with ventilation or CO detector*  *+ no contract displaying the regular maintenance of the appliance and/or flue sweeping frequency* | *There is an appliance using carbon-based fuel*  *+ no ventilation or CO detector*  *+ no contract displaying the regular maintenance of the appliance and/or flue sweeping frequency* |

|  | **Exposure to environmental airborne and soil pollution** | | | | |
| --- | --- | --- | --- | --- | --- |
|  | | **0** | **1** | **2** | **3** |
| 7. Outdoor air pollution  If there is no local strategic map or this is unknown, do not complete but  tick here☐  [See the map here](https://atmo-france.org/la-qualite-de-lair-dans-votre-region/) | | ☐ | ☐ | ☐ | ☐ |
|  |  | *Housing located in an area where the air quality standards are not exceeded*  *No agricultural activity with aerial spraying of pesticides nearby (<50m)* | *Housing located in an area where the air quality standards are potentially exceeded*  *No aerial spraying of pesticides nearby* | *Housing located in an area where the air quality standards are exceeded*  *No aerial spraying of pesticides nearby* | *Housing located in a "priority air" area*  *Or*  *Presence of agricultural activity with aerial spraying of pesticides nearby (<50 m)* |
|  |  |  |  |  |  |
| 8. Outdoor soil quality  [See the Basias map](http://www.georisques.gouv.fr/dossiers/basias/donnees%23/) [here](http://www.georisques.gouv.fr/dossiers/basias/donnees%23/) | | ☐ | ☐ | ☐ | ☐ |
|  |  | *No observed risks relating to the soil (quality, solidity)* | *Soil presenting controlled risks (treatments carried out, etc.)* | *Soil presenting risks* | *Housing built on a site listed in the BASIAS database* |

* *French Institute on Nuclear and Radiological Risk*

|  | **Noise** | | | | |
| --- | --- | --- | --- | --- | --- |
|  | | **0** | **1** | **2** | **3** |
| 9. Indoor noise nuisance | | ☐ | ☐ | ☐ | ☐ |
|  |  | *Little or no noise generated by neighbours or controlled mechanical ventilation, etc.* | *Occasional tolerable noise generated by neighbours and/or systems or networks (controlled mechanical ventilation, water evacuation, air conditioning)* | *Frequent, tolerable noise generated by neighbours and/or systems or networks and/or leading to action on the part of the occupier (mediation, complaint, sound insulation)* | *Frequent, disruptive noise generated by neighbours and/or systems or networks (e.g. in terms of sleeping or doing homework)* |
| 10. Outdoor noise nuisance | | ☐ | ☐ | ☐ | ☐ |
|  |  | *Absence of noisy activity nearby (including from traffic)* | *Presence of noisy activity only during the day, noise (including from traffic) is moderate and/or there is good sound insulation* | *Presence of noisy activity during the day and/or evening, poor sound insulation, not considered disruptive (windows closed)* | *Presence of noisy activity during the day and/or evening, poor sound insulation, considered disruptive (windows closed)* |

|  | **Lighting** | | | | |
| --- | --- | --- | --- | --- | --- |
|  | | **0** | **1** | **2** | **3** |
| 11. Outdoor light pollution | | ☐ | ☐ | ☐ | ☐ |
|  |  | *Absence of night-time outdoor light pollution + blinds/curtains/shutters provided* | *Absence of night-time outdoor light pollution, but no blinds/curtains/shutters provided*  *Or there is outdoor light pollution but blinds/curtains/shutters provided* | *There is outdoor light pollution until 22.00 + absence of or inadequate blinds/curtains/shutters provided* | *Night-time outdoor light pollution is continuous + absence of or inadequate blinds/curtains/shutters provided* |
| 12. Natural lighting of rooms | | ☐ | ☐ | ☐ | ☐ |
|  |  | *Adequate natural lighting in all rooms (e.g.: to be able to read)* | *Adequate natural lighting in living areas only* | *Inadequate natural lighting in one living area* | *Inadequate natural lighting in several living areas Or*  *No natural lighting in at least one living area* |
| 13. Artificial lighting of rooms | | ☐ | *N/A* | | ☐ |
|  |  | *Direct and adequate artificial lighting in all rooms* |  |  | *Absence of direct artificial lighting in at least one room or in hallways or stairways* |
| 14. Lighting of the housing's access areas | | ☐ | ☐ | ☐ | ☐ |
|  |  | *Adequate lighting on the entryway and relevant communal areas, with a 50 m radius span* | *Adequate lighting on the entryway and relevant communal areas, with a 10 m radius span* | *Lighting directly located only on entryway and communal areas where applicable* | *Absence of lighting of any type present on the housing quarter’s entryways* |

| **Pests** | | | | |
| --- | --- | --- | --- | --- |
|  | **0** | **1** | **2** | **3** |
| 15. Presence of pests  (cockroaches,  bed bugs,  rodents,  pigeons,  termites,  mosquitoes,  insects, etc.) | ☐ | ☐ | ☐ | ☐ |
|  | *No traces of any pest* | *No traces of any pest but presence of access routes or ducts that are not completely sealed No potential sources (waste stored nearby, larval sources, etc.)* | *No traces but presence of access routes or presence of potential sources (waste stored nearby, larval sources, etc.)* | *There are traces of pests or they are perceived as a significant nuisance* |

| **Water** | | | | |
| --- | --- | --- | --- | --- |
|  | **0** | **1** | **2** | **3** |
| 16. Wastewater evacuation system | ☐ | ☐ | ☐ | ☐ |
|  | *All facilities (sinks, basins, toilets, showers, bath tubs) are connected to a maintained collective or individual wastewater evacuation system, and/or there are no signs of malfunction.* | *Facilities are connected to a collective or individual wastewater evacuation system, but there are signs of occasional malfunction (backflow, odours by the water points)*  *Or non-controlled individual evacuation system* | *Incomplete or faulty evacuation system concerning some of the facilities (e.g.: no air trap)*  *Or recurring malfunctions* | *Absence of wastewater evacuation system* |
| 17. Drinking water | ☐ | ☐ | ☐ | ☐ |
|  | *There is a drinking water point in the housing quarters with sufficient, steady flow* | *There is a drinking water point in the housing quarters with limited flow* | *There is drinking water with risk of lead in the pipework (indoor network containing lead)* | *Absence of drinking water point in the housing quarters* |
| 18. Hot water | ☐ | ☐ | ☐ | ☐ |
|  | *There is an access point that provides sufficient hot water* | *There is an access point but water is lukewarm or flow is limited* | *There is an access point but water is lukewarm and flow is limited* | *Absence of hot water in the housing* |

|  | **Temperature conditions** | | | | |
| --- | --- | --- | --- | --- | --- |
|  | | **0** | **1** | **2** | **3** |
| 19. Heating  If N/A for the geographical area, do not complete but  tick here ☐ | | ☐ | ☐ | ☐ | ☐ |
|  |  | *There is a natural or artificial heating system* | *Natural or artificial heating system, but inadequate + perceived cold* | *Faulty natural or artificial system but presence of backup or supplementary appliances* | *Absence of heating system* |
| 20. Air conditioning  If N/A for the geographical area, do not complete but  tick here ☐ | | ☐ | ☐ | ☐ | ☐ |
|  |  | *There is a natural or artificial air conditioning system* | *Natural or artificial heating system, but inefficient in regulating a low temperature* | *Faulty natural or artificial system but presence of backup or supplementary appliances but inefficient in regulating a low temperature* | *Strong exposure to heat (e.g.: glass doors + direct sunlight, + no blinds/curtains/shutters, etc.)  Faulty natural or artificial system* |
| 21. Thermal insulation  If there is no technical diagnosis or unknown, do not complete but  tick here ☐ | | ☐ | ☐ | ☐ | ☐ |
|  |  | *EPC, A energy efficiency rating* | *EPC, BC energy efficiency rating* | *EPC, DE energy efficiency rating* | *EPC, FG energy efficiency rating* |

|  | **Indoor physical protection** | | | | |
| --- | --- | --- | --- | --- | --- |
|  | | **0** | **1** | **2** | **3** |
| *22.* Structural soundness of building (building exterior/structure)  *Roofing, frameworks, outer walls, façades, gutters, etc.*  Do not complete the table if this variable is rated 2 or 3. Report to the competent authorities at the earliest possible opportunity and  tick here ☐ | | ☐ | ☐ | ☐ | ☐ |
|  |  | *On physical inspection: all components stable and functional Protection from water runoff, infiltration and rises* | *One non-compliant component* | *Two non-compliant components* | *More than two non-compliant components* |
| 23. Structural soundness of components specific to the housing  *Flooring, ceilings, stairways, inner walls, windows, pipework, landings, etc.)*  Do not complete the table if this variable is rated 2 or 3. Report to the competent authorities at the earliest possible opportunity and  tick here ☐ | | ☐ | ☐ | ☐ | ☐ |
|  |  | *On physical inspection, all components stable and functional* | *One non-compliant component* | *Two non-compliant components* | *More than two non-compliant components* |
| 24. Safety of housing components  *Windows, balconies, stairways (railings, stairways, balconies, guardrails, etc.)*  Do not complete the table if this variable is rated 2 or 3. Report to the competent authorities at the earliest possible opportunity and  tick here ☐ | | ☐ | *N/A* | | ☐ |
|  |  | *Fall protection components and structures in good condition or no observed risks* |  |  | *Fall protection components in poor condition or no protection components on one or more structures* |
| 25. Condition of surfaces: lead risk  If housing built before 1949 and no technical diagnosis, do not complete but  tick here ☐ | | ☐ | ☐ | ☐ | ☐ |
|  |  | *Housing built after 1949* | *Housing built before 1949; lead diagnosis negative, paintwork in good condition* | *Housing built before 1949 and lead diagnosis positive / paintwork in good condition (including in the communal areas)* | *Lead diagnosis positive and paintwork chipped in the housing or communal areas* |

| 26. Natural risk protection (flooding or land instability)  [See the map here](http://www.georisques.gouv.fr/) | ☐ | ☐ | ☐ | ☐ |
| --- | --- | --- | --- | --- |
|  | *Housing located outside a risk area (flooding or land instability)* | *Housing located in a risk area*  *with low flooding risk (low probability of flooding)*  *with approved land instability risk prevention plan* | *Housing located in a risk area with management plan in place;*  *no living areas / bedroom on ground floor in a floodplain*  *geotechnical survey and protection measures in unstable land areas* | *Housing in a risk area (flooding or land instability) and no protection measures* |
| 27. Technological risk protection | ☐ | ☐ | ☐ | ☐ |
|  | *Absence of industrial plant within a 1,000m radius* | *Absence of industrial plant within a 500m radius* | *There is an industrial plant within a 500m radius* | *There are several industrial plants within a 500m radius* |
| 28. Fire protection | ☐ | ☐ | ☐ | ☐ |
|  | *Detector +* | *Detector but* | *Absence of detector and* | *Absence of detector and* |
|  | *easy evacuation* | *complex evacuation* | *complex evacuation* | *complex evacuation* |
|  |  | *(single narrow exit, high floor +* | *(single narrow exit, high floor +* | *(single narrow exit, high floor +* |
|  |  | *no window + awkward stairway, etc.)* | *no window + awkward stairway, etc.)* | *no window + awkward stairway, etc.)* |
|  |  |  |  | *(two* |
|  |  | *Or no detector and easy evacuation* | *(two unfavourable criteria)* | *unfavourable criteria)* |
|  |  | *(one of the two criteria is unfavourable)* |  | *And* |
|  |  |  |  | *Significant fire risk factors* |

| **Waste** | | | | |
| --- | --- | --- | --- | --- |
|  | **0** | **1** | **2** | **3** |
| 29. Waste collection system | ☐ | ☐ | ☐ | ☐ |
|  | *There is a waste storage space (communal bin area or storage separate from the living areas for individuals: garage, or outdoors) and regular collection* | *There is a waste storage space, but this is dilapidated* ***or*** *collection is infrequent* | *There is a waste storage space, but this is dilapidated* ***and*** *collection is infrequent* | *Absence of waste storage space* |

| **Electricity** | | | | |
| --- | --- | --- | --- | --- |
|  | **0** | **1** | **2** | **3** |
| 30. Presence of an electrical installation | ☐ | *N/A* | | ☐ |
|  | *Connection in all rooms* |  |  | *Kitchen connection insufficient to power the main household appliances* |
| 31. Condition of the electrical installation | ☐ | ☐ | ☐ | ☐ |
|  | *Condition report does not identify any non-compliance. If there is no report: no obvious risk (bare socket, bare wiring, installation manifestly old or*  *hazardous)* | *A few remarks / very low risk* | *Significant number of remarks in the report*  *If there is no condition report: observations of bare sockets or bare wiring are few and far between* | *High risks mentioned in the report*  *If there is no condition report: there are several bare sockets, bare wiring or one or more manifestly old or hazardous installations* |

|  | **Hygiene** | | | | |
| --- | --- | --- | --- | --- | --- |
|  | | **0** | **1** | **2** | **3** |
| 32. Toilets (WC) | | ☐ | ☐ | ☐ | ☐ |
|  |  | *There are serviceable toilets in the housing in a completely separate room* | *There are toilets in the housing but these are poorly separated from the living areas* | *Toilets in the housing are faulty or unusable There are toilets accessible outside of the housing* | *Absence of accessible toilets in the housing or within the rest of the building* |
| 33. Washroom | | ☐ | ☐ | ☐ | ☐ |
|  |  | *There is a serviceable, completely separate washroom in the housing, with a bath tub or shower.* | *There is a washroom in the housing but it is poorly separated from the living areas* | *There is a washroom in the housing but it is faulty or unusable*  *There is a washroom accessible outside of the housing* | *Absence of washroom with a shower or bath tub in the housing or within the rest of the building* |
| 34. Scope for cleaning | | ☐ | *N/A* | | ☐ |
|  |  | *Housing finished and includes coverings (walls and floors); in good condition, ease of cleaning guaranteed* |  |  | *Housing unfinished, building site still present, no covering over a significant surface area (wall or floor), cleaning is limited* |

|  | **Accessibility** | | | | |
| --- | --- | --- | --- | --- | --- |
|  | | **0** | **1** | **2** | **3** |
| 35. Accessibility of the housing from the outside | | ☐ | ☐ | ☐ | ☐ |
|  |  | *Housing accessible on the ground floor (no steps)* | *Housing is not on the ground floor*  *but there is an accessible lift with no steps, in working order, and appropriately sized to allow at least one wheelchair through (where there are floors) or presence of an access ramp to the building or to the entrance of the house where applicable* | *Housing is not on the ground floor There is a lift but it is out of order or inappropriately sized to allow a wheelchair through or there is no access ramp to the building or to the entrance of the house where applicable* | *Housing is not on the ground floor*  *No lift + no access ramp* |
| 36. Room size | | ☐ | *N/A* | | ☐ |
|  |  | *All rooms are satisfactory in size in terms of the surface area and ceiling height* |  |  | *At least one living area is unsatisfactory in size in terms of the surface area and ceiling height* |
| 37. Interior layout of the housing  /ease of movement + condition of surface areas | | ☐ | ☐ | ☐ | ☐ |
|  |  | *Movement inside and between rooms is very easy for everyone, with no risks of falling* | *Movement may be difficult for some frail individuals or in some rooms only, floors pose a potential risk of falling, or where some individuals are concerned* | *Movement very difficult for some frail individuals throughout the housing, floors pose a risk of falling* | *Movement very difficult between rooms and inside each room, floors pose a risk of falling where anyone is concerned* |
| 38. Accessibility inside the housing | | ☐ | ☐ | ☐ | ☐ |
|  |  | *Housing completely accessible to a person with reduced mobility* | *Housing accessible overall, and necessary changes are affordable* | *Housing accessible overall, but necessary changes will be expensive* | *Housing is not currently, nor can it be made, accessible* |
| 39. Public transport services nearby | | ☐ | ☐ | ☐ | ☐ |
|  |  | *Ample services with regular stops (several times a day or more) by public transport + accessible for everyone (including people with reduced mobility)* | *Limited services and infrequent stops (e.g.: 3 times a day or less) by public transport, but accessible for everyone (including people with reduced mobility) or good services but which are not accessible to everyone* | *Limited services and infrequent stops by public transport, not accessible for everyone* | *Absence of public transport services* |

| **Food supplies** | | | | |
| --- | --- | --- | --- | --- |
|  | **0** | **1** | **2** | **3** |
| 40. Access to good-quality food supplies nearby | ☐ | ☐ | ☐ | ☐ |
|  | *Easy access to fresh fruit and vegetables (e.g. shops less than 5 minutes away, home-grown produce)* | *Fruit and vegetable shops less than 10 minutes away* | *Absence of fruit and vegetable shops nearby (> 10 mins)* | *Absence of fruit and vegetable shops nearby + only fast-food or unhealthy food options available* |
| 41. Presence of adequate space for cooking | ☐ | ☐ | ☐ | ☐ |
|  | *Functional area for cooking* | *Kitchenette or rudimentary kitchen but where a cooking area AND refrigeration unit can be installed* | *Kitchenette or rudimentary kitchen which cannot accommodate a cooking or refrigeration area* | *Absence of kitchenette or kitchen with no drinking water* |

| **Outdoor view** | | | | |
| --- | --- | --- | --- | --- |
|  | **0** | **1** | **2** | **3** |
| 42. View outside | ☐ | ☐ | ☐ | ☐ |
|  | *View of nature* | *At least one living area overlooks another property*  *Or unrestricted view, without overlooking a nature spot* | *All rooms overlook another property* ***or*** *the main room has a windowless wall* | *All rooms overlook another property* ***and*** *the main room has a windowless wall* |

|  | **Environment conducive to exercise and socialising** | | | | |
| --- | --- | --- | --- | --- | --- |
|  | | **0** | **1** | **2** | **3** |
| 43. Green spaces | | ☐ | ☐ | ☐ | ☐ |
|  |  | *There are wide-open green spaces within the immediate vicinity* | *Small green spaces within the immediate vicinity* | *Green spaces within a 500 m radius* | *Absence of green space within a 500 m radius* |
| 44. Recreational or sports areas, communal spaces | | ☐ | ☐ | ☐ | ☐ |
|  |  | *Wide-open spaces within the immediate vicinity suitable for everyone (of all ages in particular)* | *Small spaces nearby but not suitable for everyone* | *There are spaces some distance away, but nothing easily accessible nearby* | *Absence of spaces accessible* |
| 45. Cultural activities and entertainment  *(community feast, music events, book days, etc.)* | | ☐ | ☐ | ☐ | ☐ |
|  |  | *There are cultural activities within the immediate vicinity, suitable for everyone (of all ages and affordable) as well as entertainment within the immediate vicinity* | *There are cultural activities and entertainment options within the immediate vicinity but these are not suitable for everyone* | *There is a limited choice of cultural activities and entertainment options some distance away* | *Absence of cultural activities or entertainment options* |
| 46. Cycle paths | | ☐ | ☐ | ☐ | ☐ |
|  |  | *There are dedicated footpaths or cycle paths covering a long distance (right outside the building or nearby)* | *There are dedicated cycle paths but only over a limited distance* | *There are cycle paths running alongside traffic lanes* | *Absence of cycle paths* |
| 47. Pavements | | ☐ | ☐ | ☐ | ☐ |
|  |  | *There are safely laid out pavements, clearly separated from other environmentally friendly transport routes and accessible to everyone (with benches for resting along the way, wide enough for use by a person with reduced mobility and enough space)* | *There are safely laid out pavements but they are cluttered and cannot be used safely by everyone* | *There are paved walkways, but they are not very safely laid out (not separate from the road for example), are poorly separated from other environmentally friendly transport routes and unsuitable for some people* | *Absence of pavements* |

1. **Data sources to be used for filling out each variable**


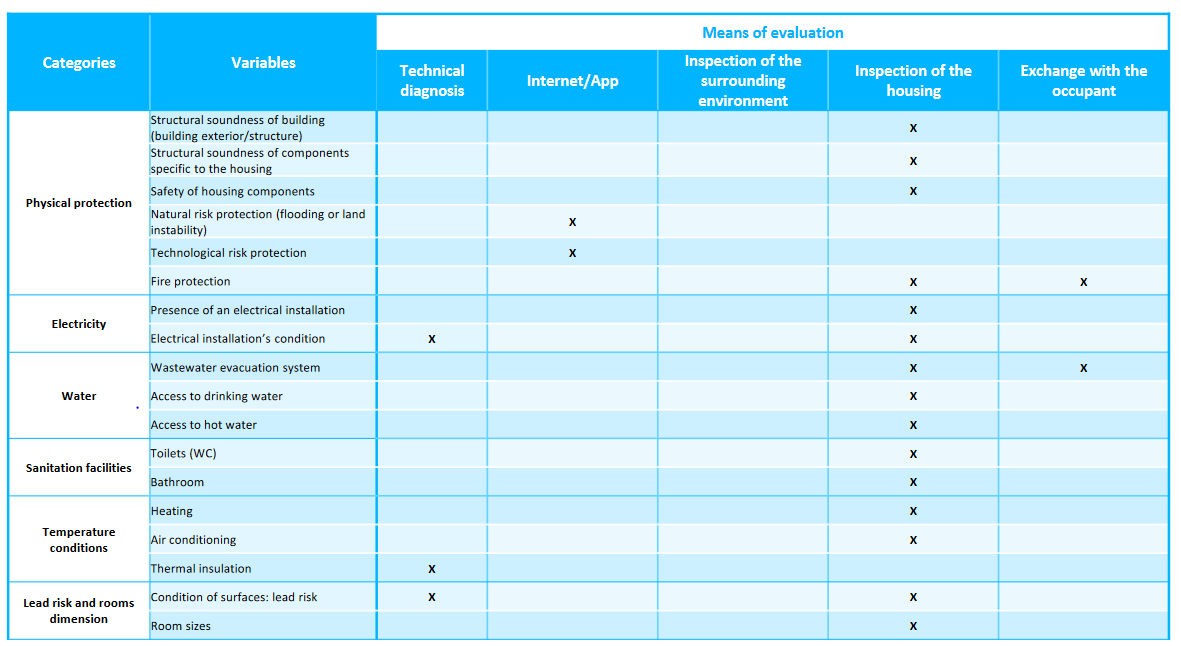

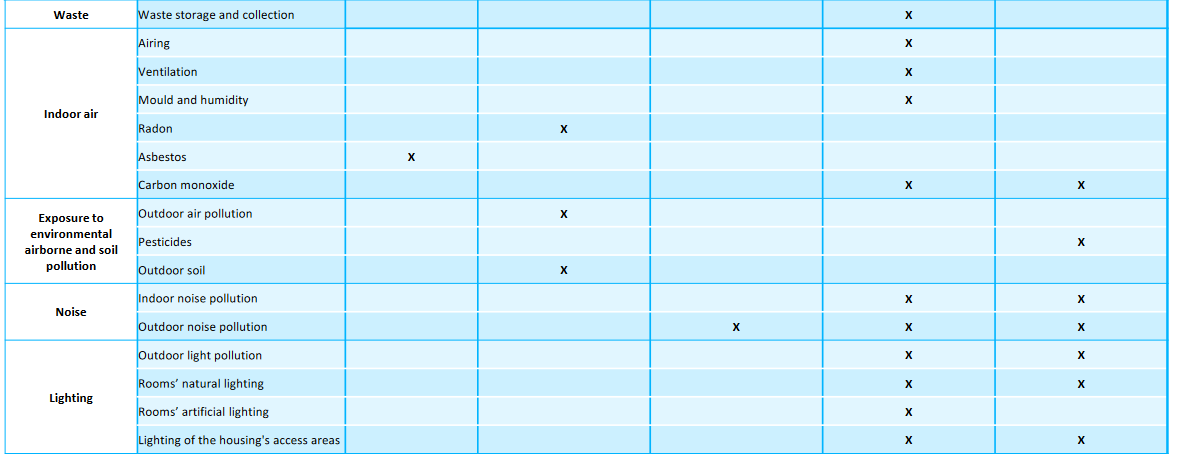

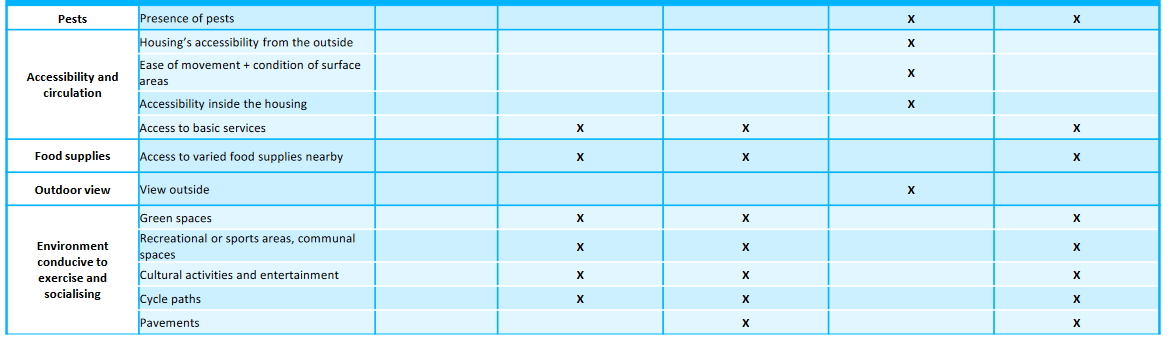


1. **Mailing lists**

Composition of the mailing lists used to recruit participants for the test period and for the public consultation, displayed as the number of emails included within the mailing lists concerning each category

| Category | Test period | Public consultation |
| --- | --- | --- |
| Real estate professional (estate agent, architect …) | - | 54 (4%) |
| Association fighting indecent housing | 6 (17%) | 10 (1%) |
| Social landlord | 9 (26%) | 209 (17%) |
| Private landlord | - | 27 (2%) |
| Social or medico-social workers | 4 (11%) | 34 (3%) |
| City service | 7 (20%) | 100 (8%) |
| State service | 2 (6%) | 529 (42%) |
| Health agency (ANSES*, Santé Publique France) | - | 3 (0%) |
| Advisor for indoor environment | 2 (6%) | 59 (5%) |
| Elected representative | 4 (11%) | 208 (16%) |
| Academic researcher | - | 24 (2%) |
| Public health association | - | 2 (0%) |
| Agglomeration | 1 (3%) | 5 (0%) |
| Network in health | - | 2 (0%) |
| TOTAL | **35** | **1266** |

*ANSES: French Agency for Food, Environmental and Occupational Health & Safety

1. **Public consultation questionnaire**

1- Are you responding to this consultation as:

- Real estate professional (estate agent, architect …)
- Association fighting indecent housing
- Social landlord
- Private landlord
- Social or medico-social workers
- City service
- State service
- Health agency (ANSES, Santé Publique France)
- Advisor for indoor environment
- Elected representative
- Academic researcher
- Other : __________

2- According to you, what advantage(s) and disadvantage(s) does a tool that characterizes a housing in regard with the occupants’ health represent? (whether it is the Domiscore or another tool you may know) *(several possible answers)*

- Provide a clear information on a property’s profile in regard with its occupants’ health
- Increase awareness on what are a property’s beneficial or detrimental characteristics in regard with health
- Enhance housing condition
- Provide a tool to inform local public policies that aim at increase housing condition and awareness
- Detect properties that are unfitted for vulnerable people
- Detect properties that are severely degraded or even hazardous and which could endanger the occupants
- Encourage an increase in the housing’s price (whether it is for rent or buy)
- Other(s): ______________________________________________

Additional comments:

3- What do you think about the structure and the different variables of the Domiscore grid?

- Understandable
- Rather understandable
- Rather difficult to understand
- Difficult to understand

*Please say why and mention the variables if relevant:*

Additional comments:

4- What do you think about the variables selected within the Domiscore grid as factors influencing health?

- Complete and relevant
- Globally relevant but some elements are lacking

*Please mention which ones:*

- Too long
- Not relevant

*Please say why and mention the variables if relevant:*

Additional comments:

5- What do you think about the scaled score assigned to each variable of the Domiscore grid?

- Relevant
- Rather relevant
- Rather not relevant
- Not relevant
  *Please say why:*

Additional comments:

6- What do you think about the Domiscore grid’s filling process?

- Easy
- Rather easy
- Rather difficult
- Difficult

*Please say why:*

Additional comments:

7- According to you, what application(s) of the Domiscore grid could be conducted by housing or city planning professionals?

- Describe a real estate condition
- Inform a tenant/owner about the health consequences of their property
- Detect problematic situations requiring an inspection by the relevant authority
- Rank granting allocated to enhance housing
- Other(s): ________________________________________

Additional comments:

Do you have any additional comment you would like to add before ending the questionnaire?

Thank you for your participation. If you wish to receive the results of the consultation, please share your email address: __________

The results bill be published on the HCSP’s website on fall 2020.

1. **Summary of participant responses to the public consultation**

| **Question** | **N (%)** |
| --- | --- |
| **2- In your opinion, what advantage(s) and disadvantage(s) does a tool that characterizes a housing in regard with the occupants’ health represent? (whether it is the Domiscore or another tool you may know) *(several possible answers)*** |  |
| **       Provide a clear information on a property’s profile in regard with its occupants’ health** | 97 (64%) |
| **       Increase awareness on what are a property’s beneficial or detrimental characteristics in regard with health** | 94 (62%) |
| **       Enhance housing condition** | 81 (54%) |
| **       Provide a tool to inform local public policies that aim to increase housing condition and awareness** | 101 (67%) |
| **       Detect properties that are unfitted for vulnerable people** | 96 (64%) |
| **       Detect properties that are severely degraded or even hazardous and which could endanger the occupants** | 109 (72%) |
| **       Encourage an increase in the housing’s price (whether it is for rent or buy)** | 8 (5%) |
| **       Do not know** | 2 (1%) |
| **3- How would you rate the structure and the different variables of the Domiscore grid?** |  |
|        **Understandable** | 56 (37%) |
|        **Rather understandable** | 84 (55%) |
|        **Rather difficult to understand** | 8 (5%) |
|        **Difficult to understand** | 3 (2%) |
| Additional comments: |  |
| Some participants commented that technical words within the grid were difficult to understand, and that some could be reworded, such as using the terms “heating system” instead of “regulating system for cold temperature”. Some participants highlighted the usefulness of the video and the links provided within the grid. |  |
| **4- How would you rate the variables selected within the Domiscore grid as factors influencing health?** |  |
|        **Complete and relevant** | 56 (37%) |
|        **Globally relevant but some elements are lacking** | 45 (30%) |
|        **Too long** | 42 (28%) |
|        **Not relevant** | 8 (5%) |
| Additional comments: |  |
| The proportion of participants who found the list too long was especially high amongst social and medico-social workers (64%); many participants, mostly from state/local services proposed to add variables (e.g. the presence of a heating device within the indoor air pollution category, the presence of a power generator, an electromagnetic field, the accessibility of health care services, the localization of the property, the relationship with the owner) or to develop some of them (waste management, electricity) and reduce others; |  |
| A couple of participants suggested to consider the view angle (roof window or wall window) within the category “outdoor view”; |  |
| One participant said the architectural style of the building should be taken into consideration (high ceiling, wooden floor etc); |  |
| One participant proposed to consider the neighborhood in terms of noise or presence of pets as well as in terms of social support it can provide; |  |
| Several participants regretted that the accessibility to Internet was not considered; |  |
| A few participants proposed to include the feeling of security of the residents, considering areas experiencing illegal traffic or violence for instance; |  |
| One participant said that the score attributed to the outdoor pollution could not be based on the website provided since it provides information about the specific day the website is consulted. |  |
| Another type of comment pointed out the need for adapting the variables/scores to the environmental settings (whether the housing is located in the city or in the countryside) and that the current version of the grid rather fitted an urban setting and assigned it a better score |  |
| **5 How would you rate the scaled scores assigned to each variable of the Domiscore grid?** |  |
|        **Relevant** | 40 (26%) |
|        **Rather relevant** | 98 (65%) |
|        **Rather not relevant** | 9 (6%) |
|        **Not relevant** | 4 (3%) |
| Additional comments: |  |
| Some participants mentioned that the score scales were sometimes unclear and subjective: |  |
| Several participants highlighted the need for rewording the scaled score in a way that is more objective, to avoid a scoring based on the assessor’s understanding of the scaled score (for example the proposed response "All rooms are satisfactory in size in terms of the surface area and ceiling height" should mention the value of an acceptable surface area and ceiling height.) |  |
| One participant mentioned that the scoring scales were overall relevant but that their terms needed to be clarified. |  |
| A few participants advocated that the absence of diagnostic should not prevent the evaluator from scoring the corresponding variable but rather give an opportunity to assess the situation in situ. |  |
| **6- How would you rate the Domiscore grid’s filling process?** |  |
|        **Easy** | 37 (21%) |
|        **Rather easy** | 87 (58%) |
|        **Rather difficult** | 29 (19%) |
|        **Difficult** | 3 (2%) |
| Additional comments: |  |
| The time needed to fill out the grid was a concern for many participants. Some workers from state or local services thought that there would not be enough time to fill the grid on their regular workday. More specifically, a few participants highlighted that the variables dealing with the outdoor environment (e.g. food stores, crops) as well as the websites and diagnostics to be consulted, were particularly time-consuming, even though they acknowledged their utility. The question of these diagnostics not always being available online was also raised. |  |
| Lack of training was another concern. A social/medico-social worker acknowledged it could be difficult for someone working in the field to correctly score housing characteristics and most of the social workers said it was difficult to understand the different websites and diagnostics. Two participants acknowledged it would be difficult for residents to actually fill out and use the grid. Others said the grid would be rather easy to fill out after having received a training beforehand, while a couple of participants mentioned the need for an explanatory note at the beginning of the grid, or even guidelines variable by variable. |  |
| **7- In your opinion, What use(s) do you imagine different housing or urban planning operators might make of the Domiscore tool (whether desirable or not)?** |  |
|        **Describe a real estate condition** | 78 (52%) |
|        **Inform a tenant/owner about the health consequences of their property** | 110 (73%) |
|        **Detect problematic situations requiring an inspection by the relevant authority** | 124 (82%) |
|        **Rank granting allocated to enhance housing** | 68 (45%) |
|        **Do not know** | 2 (1%) |
| Additional comments: |  |
| It was suggested that the Domiscore could allow an external observer to find problems with a dwelling that residents could not identify on their own; implement local awareness campaigns tailored to a housing quality mapping established using Domiscore scores; to characterize inaccessible properties; to enact expropriations; to module housing taxation; to constitute a reminder for collectivities regarding with new housing projects; to condition a building permit, condition the rent permit for a community; to support a demand for works to improve a housing condition. Several participants appreciated the awareness-raising aspect of the tool, although a participant raised concern about the anxiety-indcing potential of this long list of possible negative factors for residents. |  |

1. **Domiscore Grid with post-testing modifications**

**Domiscore Grid**

Document from the French High Council for Public Health <https://www.hcsp.fr/Explore.cgi/avisrapportsdomaine?clefr=772> Any reproduction and use of the Domiscore grid should mention its origin: French High Council for Public Health. Its text may be amended by the HCSP only.

The Domiscore is a tool that aims at evaluating a housing, based on its potential impact on the occupants’ health and well-being. It promotes healthy housing and helps identifying at-risk housing situations. It is available to every actor of the housing sector. In order to support the assessor in its evaluation, guidance on the evaluation process and the score computation is provided in the appendix. The Domiscore is easy to fill out and does not require any particular skill in terms of housing. It is anonymous and does not require the collection of information about the occupant.

**Housing’s characteristics**

**Type Profile**

Collective housing Social housing

Individual housing Private housing

**Area Occupier’s profile**

Urban area Owner-occupier

Rural area Tenant

Other: ________

**Location**

France Overseas

Metropolitan France

**Type (studio, one bedroom…):** .......................................................................................

**Region:** .............................................................................................................................

| Physical protection | | | | |
| --- | --- | --- | --- | --- |
|  | 0  (best case) | 1 | 2 | 3  (worst case) |
| 1. Physical soundness of the building (building exterior/structure)  Roofing, frameworks, outer walls, façades, gutters… |  |  |  |  |
|  | On physical inspection, all components are stable and functional + protection from water runoff, infiltration and rises | One non-compliant component  Please specify which one:  ______________________________________ | Two non-compliant components  Please specify which ones:  ___________________  ___________________ | More than two non-compliant components  Please specify which ones:  ___________________  ___________________ |
| In case of score 2 or 3, please report to the relevant authorities at the earliest possible opportunity  As well as in case of score 1 when the component is structural (roofing, frameworks, outer walls/façades) | | | | |

| 2. Structural soundness of the housing’s components  Flooring, ceilings, stairways, inner walls, windows, pipework, landings… |  |  |  |  |
| --- | --- | --- | --- | --- |
|  | On physical inspection, all components are stable and functional | One non-compliant component  Please specify which one:  __________________  __________________ | Two non-compliant components  Please specify which ones:  __________________  __________________ | More than two non-compliant components  Please specify which ones:  __________________  __________________ |
| In case of score 2 or 3, please report to the relevant authorities at the earliest possible opportunity | | | | |

| 3. Safety of the housing’s components  Windows, balconies, stairways, railings, guardrails... |  |  |  |  |  |
| --- | --- | --- | --- | --- | --- |
|  | Fall protection components and structures in good condition or no observed risks | N/A | | Fall protection components in poor condition or absence of protective components on at least one structure | |
| In case of score 2 or 3, please report to the relevant authorities at the earliest possible opportunity | | | | |  |

| 4. Protection against natural risk (flooding or land instability)  [See the map here](https://www.georisques.gouv.fr/mes-risques/connaitre-les-risques-pres-de-chez-moi) |  |  |  |  |
| --- | --- | --- | --- | --- |
|  | Housing located outside a risk area*  *flooding or land instability | Housing located in a risk area  + low probability of flooding  + approved land instability risk prevention plan | Housing located in a risk area, management plan in place  + absence of living areas/bedrooms on the ground floor in a floodplain  + geotechnical survey and protection measures in place in unstable land areas | Housing located in a risk area (flooding or land instability) and no protection measures |

| 5. Protection against technological risk  [See the map here](https://www.georisques.gouv.fr/mes-risques/connaitre-les-risques-pres-de-chez-moi) |  |  |  |  |
| --- | --- | --- | --- | --- |
|  | Housing located outside a technological risk prevention plan’s perimeter  +  Absence of industrial plant within a 1,000m radius, industrial plant releasing pollutants within a 5,000m radius and polluted or potentially polluted sites within a 500m radius | Housing located outside a technological risk prevention plan’s perimeter  +  Presence of industrial plant within a 1,000m radius or industrial plant releasing pollutants within a 5,000m radius or polluted or potentially polluted sites within a 500m radius | Housing located within a technological risk prevention plan’s perimeter | Housing located within a technological risk prevention plan’s perimeter  +  Presence of polluted or potentially polluted sites within a 500m radius |

| 6. Fire protection |  |  |  |  |
| --- | --- | --- | --- | --- |
|  | The housing is equipped with a smoke detector  +  It can be easily and safely evacuated and has a fire containment capacity  +  The building is easily accessible to emergency devices and vehicles  +  The housing does not present any of the following fire risk factors: non-maintained chimney, faulty ventilation system, expired gas pipe | The housing is equipped with a smoke detector  +  It can be easily and safely evacuated and has a fire containment capacity  +  The building is easily accessible to emergency devices and vehicles  +  The housing presents one of the following fire risk factors: non-maintained chimney, faulty ventilation system, expired gas pipe | The housing is equipped with a smoke detector  But presents one of the following features:  - Poorly accessible to emergency devices and vehicles  - Complex evacuation (only one exit, narrow and not protected, no window…)  - Absence of circuit-breaker or presence of bare wires  - Improper connections (supply and evacuation) of gas devices | The housing is not equipped with a smoke detector  or  The housing is equipped with a smoke detector  But presents one of the following features:  - Poorly accessible to emergency devices and vehicles  - Complex evacuation (only one narrow and not protected exit, no window…)  - Absence of circuit-breaker or presence of bare wires  - Improper connections (supply and evacuation) of gas devices |
| In case of score 2 or 3, please report to the relevant authorities at the earliest possible opportunity | | | | |

**Category’s score:**

| Electricity | | | | |
| --- | --- | --- | --- | --- |
|  | 0  (best case) | 1 | 2 | 3  (worst case) |
| 7. Presence of an electrical installation |  |  |  |  |
|  | Connection in all rooms | N/A | | Kitchen connection insufficient to power the main household appliances |
| In case there is no electrical installation, or in case of score 3, please report to the relevant authorities at the earliest possible opportunity | | | | |

| 8. Electrical system’s condition |  |  |  |  |
| --- | --- | --- | --- | --- |
|  | The diagnostic report does not identify any non-compliance  If there is no report: no obvious risk (bare socket, bare wires, manifestly old or hazardous installation) | N/A | Low risks mentioned in the diagnostic report  If there is no diagnostic report: observation of rare bare sockets or bare wires | High risks mentioned in the report  If there is no diagnostic report: observation of several bare sockets, bare wiring or one or more manifestly old or hazardous installations |
| In case of score 3, please report to the relevant authorities at the earliest possible opportunity | | | | |

**Category’s score :**

| Water | | | | |
| --- | --- | --- | --- | --- |
|  | 0  (best case) | 1 | 2 | 3  (worst case) |
| 9. Wastewater evacuation system |  |  |  |  |
|  | All facilities (sinks, basins, toilets, showers, bath tubs) are connected to a maintained and functional collective or individual* wastewater evacuation system  *not connected to the sewage system | All facilities are connected to a collective or individual wastewater evacuation system, but there are signs of occasional malfunction (backflow, odors by the water points)  or  Non-controlled individual evacuation system | Incomplete or faulty evacuation system concerning some of the facilities (e.g.: no air trap)  or  Wastewater system with recurring malfunctions | Absence of wastewater evacuation system with backflow in outdoor environments |
| In case of score 3, please report to the relevant authorities at the earliest possible opportunity | | | | |

| 10. Access to drinking water |  |  |  |  |
| --- | --- | --- | --- | --- |
|  | There is a drinking water access point in every water room | There is a drinking water access point in some water rooms | There is only one drinking water access point within the housing | Absence of drinking water point within the housing |
| In case of score 3, please report to the relevant authorities at the earliest possible opportunity | | | | |

| 11. Access to hot water |  |  |  |  |
| --- | --- | --- | --- | --- |
|  | There is a hot water access point in the bathroom and kitchen | There is a hot water access point in the bathroom and kitchen but water is lukewarm | There is a hot water access point in the bathroom or the kitchen | Absence of hot water access point within the housing |
| In case of score 3, please report to the relevant authorities at the earliest possible opportunity | | | | |

**Category’s score :**

| Sanitation facilities | | | | |
| --- | --- | --- | --- | --- |
|  | 0  (best case) | 1 | 2 | 3  (worst case) |
| 12. Toilets (WC) |  |  |  |  |
|  | There are functional toilets in the housing in a completely separate room | There are functional toilets in the housing but these are poorly separated from the living areas or from the kitchen | Toilets in the housing are faulty or unusable | There are toilets in the housing but these are poorly separated from the living areas or from the kitchen |
| In case of score 3, please report to the relevant authorities at the earliest possible opportunity | | | | |

| 13. Bathroom |  |  |  |  |
| --- | --- | --- | --- | --- |
|  | There is a functional, completely separate bathroom in the housing, with a bath tub or a shower | There is a bathroom in the housing but it is poorly separate from the living areas | There is a bathroom in the housing but it is faulty or unusable | There is a functional, completely separate bathroom in the housing, with a bath tub or shower |

**Category’s score:**

| Temperature conditions | | | | |
| --- | --- | --- | --- | --- |
|  | 0  (best case) | 1 | 2 | 3  (worst case) |
| 14. Heating  If not relevant regarding with the geographical area, do not complete but tick here |  |  |  |  |
|  | There is a functional and sufficient natural* or artificial heating system  *thermic insulation, window protection, Nordic well | There is a functional natural or artificial heating system, but insufficient + perceived cold | Faulty natural or artificial heating system + perceived cold | Absence of heating system + perceived cold |
| In case of score 3, please report to the relevant authorities at the earliest possible opportunity | | | | |

| 15. Cooling system  If not relevant regarding with the geographical area, do not complete but tick here |  |  |  |  |
| --- | --- | --- | --- | --- |
|  | There is a natural* or artificial air conditioning system  *air-ground heat exchanger, window shutters, possible night ventilation | There is a functional natural or artificial conditioning system, but insufficient + perceived heat | Faulty natural or artificial conditioning system + perceived heat | Absence of natural or artificial conditioning system + perceived heat |

| 16. Thermal insulation  If there is no technical diagnosis or unknown, do not complete but tick here |  |  |  |  |
| --- | --- | --- | --- | --- |
|  | EPC, A/B energy efficiency rating | EPC, C energy efficiency rating | EPC, D/E energy efficiency rating | EPC, F/G energy efficiency rating |

**Category’s score:**

| Lead risk and rooms’ size | | | | |
| --- | --- | --- | --- | --- |
|  | 0  (best case) | 1 | 2 | 3  (worst case) |
| 17. Condition of surfaces: lead risk  If housing built before 1949 and no technical diagnosis, do not complete but tick here |  |  |  |  |
|  | Housing built after 1975  or  Housing with a negative lead diagnosis | Housing built before 1975  + no available diagnosis  + paintwork in good condition | Housing built before 1975  + positive lead diagnosis  + paintwork in good condition (including in the communal areas) | Positive lead diagnosis  + chipped paintwork in the housing or in the communal areas |
| In case of score 2 or 3, or if the housing was built before 1949 but there is no technical diagnosis, please report to the relevant authorities at the earliest possible opportunity | | | | |

| 18. Rooms’ size  If there is no technical diagnosis or unknown, do not complete but tick here |  |  |  |  |
| --- | --- | --- | --- | --- |
|  | There is at least one room with a minimum surface of 9m², a minimum ceiling height of 2.50m and a minimum living volume of 22.5m^3^ | There are several rooms with a minimum surface of 9m², a minimum ceiling height of 2.20m and a minimum living volume of 20m^3^ | There is one room with a minimum surface of 9m², a minimum ceiling height of 2.20m and a minimum living volume of 20m^3^ | There is no room with a minimum surface of 9m², a minimum ceiling height of 2.20m and a minimum living volume of 20m^3^ |
| In case of score 3, please report to the relevant authorities at the earliest possible opportunity | | | | |

**Category’s score:**

| Waste | | | | |
| --- | --- | --- | --- | --- |
|  | 0  (best case) | 1 | 2 | 3  (worst case) |
| 19. Waste storage and collection |  |  |  |  |
|  | There is a waste storage space* and regular collection  *communal bin area or storage separated from the living areas for individual housing (garage or outdoors) | There is a waste storage space, but it is not maintained  or  Collection is unfrequent (based on the area’s usual collection frequency) | There is a waste storage space, but it is not maintained  and  Collection is unfrequent (based on the area’s collection frequency) | Absence of waste storage space or absence of collection |

**Category’s score:**

| Indoor air | | | | |
| --- | --- | --- | --- | --- |
|  | 0  (best case) | 1 | 2 | 3  (worst case) |
| 20. Airing |  |  |  |  |
|  | There are easy-to-open windows in all living areas | There are windows but their opening is limited because of a slight obstruction or a malfunction in at least one of the living areas | There are windows but their opening is impossible because of an obstruction or a malfunction in at least one of the living areas | Absence of windows in any or some of the living areas |

| 21. Ventilation |  |  |  |  |
| --- | --- | --- | --- | --- |
|  | There is a functional continuous mechanical or natural ventilation* in all water rooms  *operationally controlled mechanical ventilation system, securely placed base-level and above-level air inlets | Absence of ventilation in one of the water rooms | Absence of ventilation in all water rooms | Absence of ventilation |

| 22. Mould and humidity |  |  |  |  |
| --- | --- | --- | --- | --- |
|  | Absence of humidity | Presence of humidity but absence of mould (excluding windows or door seals) | Presence of mould in water rooms only, or presence of mould between 0.2m² and 1m² in living areas (excluding windows or door seals) | Presence of mould on a surface superior to 1m² in all rooms (excluding windows or door seals) |

| 23. Radon  [See the map here](https://www.georisques.gouv.fr/mes-risques/connaitre-les-risques-pres-de-chez-moi) |  |  |  |  |
| --- | --- | --- | --- | --- |
|  | Measured radon values below 100Bq/m^3^  or  Municipality referenced 1 by the IRSN* (low risk)  or  Municipality referenced 2 or 3 by the IRSN* (low risk) + elevated housing  * French Institute on Nuclear and Radiological risk | Measured radon values between 100 and 300Bq/m^3^  or  Municipality rated 2 by the IRSN (potential risk) + housing on the ground floor | Measured radon values between 300 and 1000Bq/m^3^  or  Municipality rated 3 by the IRSN (high risk) + housing that is underground, semi-buried or on the ground floor + system enabling the evacuation of radon in the sub-soil | Measured radon values above 1000Bq/m^3^  or  Municipality rated 3 by the IRSN (high risk) + housing that is underground, semi-buried or on the ground floor + no known evacuation system |

| 24. Asbestos  If there is no technical diagnosis or if unknown, do not complete but tick here |  |  |  |  |
| --- | --- | --- | --- | --- |
|  | Absence of asbestos in the housing (nor communal areas when appropriate) | Absence of asbestos in the housing, but presence in the communal areas with periodic assessment required | Presence of asbestos in the housing and periodic assessment required | Presence of asbestos in the housing and recommended or required removal |

| 25. Carbon monoxide |  |  |  |  |
| --- | --- | --- | --- | --- |
|  | Absence of appliance using a carbon-based fuel (wood pellets, coal, gas, oil…) | There is a gas cooker  or  There is an appliance (other than a gas cooker) using carbon-based fuel-fired with good ventilation or CO detector  + contract bearing the appliance's maintenance and/or the flue sweeping frequency | There is an appliance (other than a gas cooker) using carbon-based fuel with ventilation or CO detector  + absence of contract displaying the regular maintenance of the appliance and/or the flue sweeping frequency | There is an appliance using carbon-based fuel  + no ventilation or CO detector  + no contract displaying the regular maintenance of the appliance and/or the flue sweeping frequency |

**Category’s score:**

| Exposure to environmental airborne and soil pollution | | | | |
| --- | --- | --- | --- | --- |
|  | 0  (best case) | 1 | 2 | 3  (worst case) |
| 26. Outdoor air pollution  [See the map here](https://www.georisques.gouv.fr/mes-risques/connaitre-les-risques-pres-de-chez-moi) (the annual index should be considered)  If there is no local strategic map or this is unknown, do not complete but tick here |  |  |  |  |
|  | Housing located in an area with a “very good” air quality score (1 or 2) | Housing located in an area with a “good” air quality score (3 or 4) | Housing located in an area with a “medium” or “poor” air quality score (5, 6 or 7) | Housing located in an area with a “bad” or “very bad” air quality score (8, 9 or 10) |

| 27. Pesticides |  |  |  |  |
| --- | --- | --- | --- | --- |
|  | No agricultural activity with aerial spraying of pesticides within a radius below 500m | There is an agricultural activity with aerial spraying of pesticides within a radius between 500m and 50m | There is an agricultural activity with aerial spraying of pesticides within a radius between 10 and 50m | There is an agricultural activity with aerial spraying of pesticides within a radius below 10m |

| 28. Outdoor soil  [See the map here](https://www.georisques.gouv.fr/mes-risques/connaitre-les-risques-pres-de-chez-moi) |  |  |  |  |
| --- | --- | --- | --- | --- |
|  | No observed risks relating to the soil (pollution, solidity) | Soil presenting controlled risks (e.g. treatments carried out) | Soil presenting some risks | Housing built on a site referenced in the BASIAS database |

**Category’s score:**

| Noise | | | | |
| --- | --- | --- | --- | --- |
|  | 0  (best case) | 1 | 2 | 3  (worst case) |
| 29. Indoor noise pollution*  *Noise generated by the neighbours, appliance or networks (controlled mechanical ventilation, conditioner, waste water evacuation system) |  |  |  |  |
|  | Absence of noise | Occasional tolerable noise | Frequent noise causing an important discomfort (e.g. in terms of sleeping or doing homework) | Frequent noise causing an important discomfort (e.g. in terms of sleeping or doing homework) that led to an action from the occupier (mediation, complaint, insulation works considered) |

| 30. Outdoor noise pollution*  *Traffic is considered |  |  |  |  |
| --- | --- | --- | --- | --- |
|  | Absence of noisy activities nearby | Noisy activities during the day, moderate noise and/or good sound insulation, no discomfort (when windows are closed) | Noisy activities during the day, evening or night, poor sound insulation, no discomfort (when windows are closed) | Noisy activities during the day, evening or night, poor sound insulation, causing a discomfort (when windows are closed) |

**Category’s score:**

| Lighting | | | | |
| --- | --- | --- | --- | --- |
|  | 0  (best case) | 1 | 2 | 3  (worst case) |
| 31. Outdoor light pollution |  |  |  |  |
|  | Absence of night-time outdoor light pollution + possibility of occultation | Absence of night-time outdoor light pollution, but no possibility of occultation  or  There is outdoor light pollution + possibility of occultation | There is night-time outdoor light pollution + absence of or insufficient possibility of occultation | Night-time outdoor light pollution is continuous + absence of or insufficient possibility of occultation |

| 32. Rooms’ natural lighting |  |  |  |  |
| --- | --- | --- | --- | --- |
|  | Sufficient natural lighting in all rooms (e.g. to be able to read) | Sufficient natural lighting in living areas | Insufficient natural lighting in one living area | Insufficient natural lighting in several living areas  or  Absence of natural lighting in at least one living area |

| 33. Rooms’ artificial lighting |  |  |  |  |
| --- | --- | --- | --- | --- |
|  | Direct and sufficient artificial lighting in all rooms | N/A | | Absence of direct artificial lighting in at least one room or in hallways or stairways |

| 34. Lighting* of the housing's access areas  *permanent or only when passing by |  |  |  |  |
| --- | --- | --- | --- | --- |
|  | Sufficient lighting from the road to the entryway, on the front door access and on the front door (and communal areas when relevant) | Sufficient lighting on the front door access and on the front door (and communal areas when relevant) but not on the road to access the structure | Sufficient lighting on the front door (and communal areas when relevant) but not on the road to access the structure nor on the front door access | Absence of lighting on the housing’s entryways |

**Category’s score:**

| Pests | | | | |
| --- | --- | --- | --- | --- |
|  | 0  (best case) | 1 | 2 | 3  (worst case) |
| 35. Presence of pests  Cockroaches, bed bugs, rodents, termites,mosquitoes, insects… |  |  |  |  |
|  | Absence of pests + absence of access routes or ducts that are not completely sealed + absence of potential sources (waste storage nearby, breeding place...)  *observed by the evaluator or mentioned by the occupier | Absence of pests but presence of access routes or ducts that are not completely sealed  + absence of potential sources (waste storage nearby, breeding place...) | Absence of pests but presence of access routes or ducts that are not completely sealed  + presence of potential sources (waste storage nearby, breeding place...) | Presence of pests is obvious (traces, excrement, underground gallery, corpse, nest) or important discomfort perceived by the occupants |

**Category’s score:**

| Accessibility and circulation | | | | |
| --- | --- | --- | --- | --- |
|  | 0  (best case) | 1 | 2 | 3  (worst case) |
| 36. Housing’s accessibility from the outside |  |  |  |  |
|  | Housing on the ground floor (no steps)  or  Elevated housing with a functional elevator accessible without step whose size allows at least one wheeling chair through  And presence of an access ramp to the structure | Housing on the ground floor but absence of an access ramp to the structure  or  Elevated housing whose functional elevator does not allow at least one wheeling chair through | Elevated housing whose elevator is faulty | Elevated housing without elevator |

| 37. Ease of movement + condition of surface areas |  |  |  |  |
| --- | --- | --- | --- | --- |
|  | Movement inside and between rooms is very easy for everyone, with no risks of falling | Movement may be difficult for some frail individuals or in some rooms only, floors pose a potential risk of falling or where some individuals are concerned | Movement is very difficult for some frail individuals throughout the housing, floors pose a risk of falling | Movement is very difficult between rooms and inside each room, floors pose a risk of falling where anyone is concerned |

| 38. Accessibility inside the housing |  |  |  |  |
| --- | --- | --- | --- | --- |
|  | Housing completely accessible to a person with reduced mobility | Housing accessible overall, and necessary changes are affordable | Housing accessible overall, but necessary changes will be expensive | Housing is not currently, nor can it be made, accessible |

| 39. Access to basic services  Physician, drug store, school, post office |  |  |  |  |
| --- | --- | --- | --- | --- |
|  | Basic services are accessible in less than 10 minutes*  *by foot, public transport or car | Basic services are accessible between 10 and 20 minutes | Basic services are accessible between 20 and 30 minutes | Basic services are not accessible in less than 30 minutes |

**Category’s score:**

| Food supplies | | | | |
| --- | --- | --- | --- | --- |
|  | 0  (best case) | 1 | 2 | 3  (worst case) |
| 40. Access to varied food supplies nearby |  |  |  |  |
|  | Access point to fresh fruits and vegetables in less than 5 minutes* or home-grown products  *by foot, public transport or car | Access point to fresh fruits and vegetables in less than 10 minutes | Absence of access point to fresh fruits and vegetables in less than 10 minutes | Absence of access point to fresh fruits and vegetables in less than 10 minutes  + only fast-food or unhealthy ready to eat products (industrial cookies, sandwiches, or meal to reheat, French fries, sodas...) are available |

**Category’s score:**

| Outdoor view | | | | |
| --- | --- | --- | --- | --- |
|  | 0  (best case) | 1 | 2 | 3  (worst case) |
| 41. View outside |  |  |  |  |
|  | View of nature | At least one room overlooks another property  or  Unrestricted view, without overlooking a nature spot | All rooms overlook another property  or  The main room has a windowless wall | All rooms overlook another property  and  The main room has a windowless wall |

**Category’s score:**

| Environment conducive to exercise and socializing | | | | |
| --- | --- | --- | --- | --- |
|  | 0  (best case) | 1 | 2 | 3  (worst case) |
| 42. Green spaces |  |  |  |  |
|  | There are wide-open green spaces accessible in less than 5 minutes by foot | There are small green spaces accessible in less than 5 minutes by foot | There are green spaces accessible between 5 and 10 minutes by foot | Absence of green spaces accessible in less than 10 minutes by foot |

| 43. Recreational or sports areas, communal spaces  Cafés, public squares, sport halls, playground… |  |  |  |  |
| --- | --- | --- | --- | --- |
|  | There are accessible wide-open spaces at less than 5 minutes suitable for everyone (of all ages in particular) | There are accessible small spaces at less than 5 minutes but not suitable for everyone | There are spaces at more than 10 minutes, but poorly accessible and not suitable for everyone | Absence of accessible spaces |

| 44. Cultural activities and entertainment  Cinema, theatre, library, community feast, music events, book days… |  |  |  |  |
| --- | --- | --- | --- | --- |
|  | There are cultural activities accessible in less than 10 minutes* and suitable for everyone (of all ages and affordable)  *by foot, public transport or car | There are cultural activities accessible in less than 10 minutes but these are not suitable for everyone | There is a limited choice of cultural activities and entertainment options accessible in more than 10 minutes | Absence of cultural activities or entertainment options |

| 45. Cycle paths |  |  |  |  |
| --- | --- | --- | --- | --- |
|  | There are safe roads  or  There are dedicated cycle paths covering a long distance, right outside the building or nearby | There are dedicated cycle paths but only over a limited distance, right outside the building or nearby | There are shared cycle paths, right outside the building or nearby | Absence of safe roads  or  Absence of cycle paths right outside the building or nearby |

| 46. Pavements |  |  |  |  |
| --- | --- | --- | --- | --- |
|  | There are safely laid out pavements, clearly separated from other environmentally friendly transport routes and accessible to everyone (with benches for resting along the way, wide enough to be used by a person with reduced mobility and safe flooring) | There are safely laid out pavements but they are cluttered and cannot be used safely by everyone | There are paved walkways, but they are not very safely laid out (not separate from the road for example), are poorly separated from other environmentally friendly transport routes and unsuitable for some people | Absence of pavements |

**Category’s score:**

| Grey highlighted variables’ scores can be penalized in case of vulnerable occupants (guidance provided in the appendices) |
| --- |

**Scores summary table**

| Category | Score |
| --- | --- |
| Physical protection |  |
| Electricity |  |
| Water |  |
| Sanitation facilities |  |
| Temperature conditions |  |
| Lead risk and rooms’ size |  |
| Waste |  |
| Indoor air |  |
| Exposure to environmental airborne and soil pollution |  |
| Noise |  |
| Lighting |  |
| Pests |  |
| Accessibility and circulation |  |
| Food supplies |  |
| Outdoor view |  |
| Environment conducive to exercise and socializing |  |
| Overall score |  |

**Annex G: French regulatory framework for safe housing**

By law, any dwelling is said to be substandard (*indigne* in French) when it is either indecent (*indécent*), unhealthy (*insalubre*) or in danger (*en péril*) and does not meet the minimum set standards of habitability (37). These minimum set standards of habitability are defined by the departmental sanitary regulations, adapted from a 1978 national sanitary regulation established by the French public health code. Furthermore, several diagnostics are mandatory in the case of sale or rental, namely regarding energetic performance, lead, asbestos, radon, pests, electricity, gas, non-collective sanitation, as well as natural risks (i.e risk of flooding or landslide) (38). Funding is available to help with renovations, such as financial support provided by the National Agency for Housing (*Agence Nationale de l’Habitat,* or *ANAH*), the energy allowance (*prime énergie*) for renovations reducing energy waste (39,40), along with private contributions (foundations, NGOs).

The French High Council for Public Health (HCSP)

The HCSP is a public and independent organism that responds to formal requests from ministries and parliament committees to advise them on public health policy making (e.g. prevention, health security health system performance, etc.). The HCSP brings together nearly 80 independent voluntary experts highly qualified in various disciplines of which renowned specialists of the medical field, working as college teachers, doctors in university hospitals or researchers in public labs (41).
